# Comparative analysis of RT-qPCR, flow cytometry, and Di-4-ANEPPDHQ fluorescence for distinguishing macrophages phenotypes

**DOI:** 10.1016/j.bbrep.2025.102225

**Published:** 2025-08-30

**Authors:** Ahmed Abu Siniyeh, Walhan Alshaer, Nirmeen Elzogheir, Majed Al-Holi, Dana A. Alqudah, Duaa Abuarqoub, Joanna M. Kwiatek

**Affiliations:** a Department of Medical Laboratory Sciences, School of Science, The University of Jordan, Amman, Jordan; b Cell Therapy Center, The University of Jordan, Amman, Jordan; c Faculty of Pharmacy and Medical Sciences, University of Petra, Amman, Jordan; d Polonium Foundation, Warszawa, Poland

**Keywords:** Macrophages, M1/M2 polarization, RT-qPCR, Flow cytometry, Di-4-ANEPPDHQ fluorescence, Membrane order

## Abstract

This study evaluates the effectiveness of fluorescence microscopy using Di-4-ANEPPDHQ in differentiating macrophage phenotypes (M0, M1, and M2) compared to RT-qPCR and flow cytometry. Using THP-1 monocyte-derived macrophages, we assessed cytokine expression (IL-1β, IL-6, IL-10) via RT-qPCR, surface markers (CD86, CD64, CD206) through flow cytometry, and membrane properties with Di-4-ANEPPDHQ fluorescence. RT-qPCR showed significant differences in cytokine expression: M1 macrophages had elevated IL-1β (p < 0.0001) and IL-6 (p < 0.0001), while M2 macrophages exhibited higher IL-10 levels (p = 0.0030). Flow cytometry revealed distinct surface marker profiles, with M1 expressing high CD64 and M2 showing increased CD206. Di-4-ANEPPDHQ fluorescence indicated membrane order differences: M1 macrophages were depolarized (red shift), while M2 macrophages were hyperpolarized (blue shift). Statistical analysis confirmed high sensitivity and specificity for RT-qPCR and flow cytometry, while Di-4-ANEPPDHQ fluorescence technique provides real-time observations of changes in macrophage membrane behavior, enhancing understanding of their dynamic properties under various conditions. These findings highlight the value of integrating these methods for comprehensive macrophage phenotype characterization, which can aid in understanding macrophage polarization in immune responses and disease contexts.

## Highlights

• Di-4-ANEPPDHQ fluorescence differentiates macrophage phenotypes via membrane order.
• RT-qPCR confirms cytokine expression shifts in M1 and M2 macrophages.
• Flow cytometry validates surface markers for M1 (CD64) and M2 (CD206) phenotypes.
• Fluorescence imaging reveals M1 depolarization and M2 hyperpolarization (p < 0.0001).
• Integrating these methods enhances macrophage characterization and therapeutic insights.

## 1 Introduction

Macrophages are multifunction immune cells that play fundamental tasks in the immune response, showing a range of functional states typically categorized into three phenotypes: M0, M1, and M2. M0 macrophages are in a resting state, maintaining baseline activity and responsiveness to stimuli [1]. After activation by pro-inflammatory signals, such as interferon-gamma (IFN-γ) and lipopolysaccharides (LPS), M0 macrophages differentiate into M1 macrophages, which are associated with pro-inflammatory responses and produce cytokines like tumor necrosis factor-alpha (TNF-α) and interleukin-6 (IL-6), along with reactive species [2]. On the other side, anti-inflammatory signals like IL-4 and IL-13 originate M2 macrophages promoting tissue repair and resolution of inflammation [3,4]. In addition, they secrete anti-inflammatory cytokines, such as IL-10, and play roles in wound healing and suppression of excessive inflammation [5].

Conventional methods for differentiating macrophage phenotypes, such as gene expression analysis and surface marker profiling, based on functional and molecular profiles. Low levels of pro-inflammatory cytokines is the hallmark of M0 macrophages which distinguish them from their M1 counterparts [6]. Pro-inflammatory cytokines like TNF-α, IL-1β, and IL-6, are highly expressed by M1 Macrophages, whereas anti-inflammatory cytokines such as IL-10 and TGF-β, alongside markers like CD206 are exhibited by M2 macrophages [7,8]. In this matter, techniques like qPCR and ELISA measure these cytokine and gene expression profiles [9,10].

Surface markers are usually analyzed by flow cytometry and immunohistochemistry, with higher levels of CD86 and CD64 expression in M1 macrophages, revealing their pro-inflammatory role, while M2 macrophages show CD163, CD206, and CD200R expression [11,12]. On the other hand, baseline expression levels of markers like CD68 are normally made by M0 Macrophages [13]. These subsets are quantified and identified by Fluorescence-activated cell sorting (FACS) [14,15].

Advanced imaging techniques enable real-time tracking of macrophages are the substitution of traditional markers as they do not fully expose macrophage dynamics *in vivo* [16]. Optical imaging techniques, including bioluminescence imaging (BLI) and fluorescence imaging, offer additional experiences, although BLI suffers from poor spatial resolution and fluorescence imaging is limited to superficial tissues [17,18]. In overall, imaging techniques collectively boost and improve the understanding of macrophage behavior in physiological and pathological contexts.

In this context, by measuring membrane order of macrophages, fluorescence microscopy with Di-4-ANEPPDHQ offers an advantageous approach for distinguishing macrophage phenotypes. Di-4-ANEPPDHQ, a voltage-sensitive dye, could determine macrophages membrane order by displaying fluorescence shifts when bound to lipid bilayers, reflecting membrane potential changes [19,20]. Previous studies have demonstrated that macrophage activation is accompanied by significant changes in membrane lipid order. For example, Goossens et al. (2019) used Di-4-ANEPPDHQ to show that inflammatory macrophages exhibit disrupted membrane domains and impaired lipid raft integrity, which in turn affect immune signaling pathways [21]. These findings support the relevance of using membrane-order-sensitive probes such as Di-4-ANEPPDHQ in macrophage research. This study aims to improve macrophage characterization by integrating Di-4-ANEPPDHQ fluorescence with RT-qPCR and flow cytometry, to better understand macrophage activation dynamics. Correct identification of macrophage subsets is necessary for developing targeted therapies, as modulating polarization may optimize and improve treatment outcomes [22].

## 2 Methods

### 2.1 Cell culture

Monocyte cell line THP-1 was obtained from the American Type Culture Collection (ATCC). Cultured using RPMI 160 media (Gibco, USA), supplemented with 20 % (v/v) fetal bovine serum (FBS), 4.5 g/L d-glucose, 1 % l-Glutamine, and 1 % penicillin/streptomycin. The cells were incubated at 37 °C in a humidified incubator with 5 % CO2. The cells were sub-cultured every three days.

### 2.2 Differentiation and polarization

To differentiate M0 macrophages from THP-1 monocytes, cells were incubated with 150 nM phorbol 12-myristate 13-acetate (PMA) for 24 h, followed by 24 h in fresh medium. M1 polarization was induced by treating cells with 100 ng/mL lipopolysaccharide (LPS) and 20 ng/mL interferon-gamma (IFN-γ) for 72 h. M2 polarization was achieved by treating cells with 20 ng/mL interleukin-4 (IL-4) and 20 ng/mL interleukin-13 (IL-13) for 72 h.

### 2.3 RT-qPCR

THP-1 cells were seeded in a 6-well plate, 1 × 10^6^ cells/well. They differentiated and polarized to M0, M1, and M2 cells, respectively. The triazole-hybrid method was used to lyse the cells, and then the RNeasy Plus Mini Kit column (Qiagen, USA) was used for the RNA extraction. To quantify the extracted RNA, Nanodrop was used Nanodrop (Thermo Fisher Scientific). To synthesize cDNA, 1000 ng of total RNA was used with the RT master mix (Takara, Japan) according to manufacturer protocol. The reverse transcription was performed on a thermal cycler (Thermo Fisher Scientific, USA). RT-PCR was performed on CFX96C1000 Touch thermal cycler (Bio-Rad, CA, USA) as follows: each 10 μL qPCR reaction consisted of 2 μL cDNA, 0.2 μL (200 nM final concentration) forward primer, 0.2 μL (200 nM final concentration) reverse primer, 2.6 μL nuclease-free water, and 5 μL qPCR Master Mix (Promega, USA). The following temperature settings were used for the qPCR run: (1) 95 °C for 3 min, (2) 40 cycles of 95 °C for 5 s, and 61 °C for 30 s. Temperature settings for the melting curve: (1) 65 °C for 0:05 min, (2) 95 °C for 0:5 min. The reference gene was 18 S rRNA. Primer efficiencies were validated using standard curves generated from serial dilutions of pooled cDNA. All primer pairs exhibited amplification efficiencies between 95 % and 101 %, allowing for accurate use of the 2^–ΔΔCq method for fold-change analysis. All qPCR experiments were performed in triplicate using three independent biological replicates (n = 3). Each replicate consisted of separately cultured, differentiated, and polarized THP-1 cells.

### 2.4 Flow cytometry

To investigate the differentiation and polarization, 1.25 × 10^6^ cells/well of THP-1 cells were seeded in a 6-well plate. After the differentiation and polarization, M0, M1, and M2 macrophages were detached using 1 mL/well of accutase and centrifuged at 200×*g* for 5 min. THP-1 cells were collected by centrifugation at 200×*g* for 5 min. After washing the cells with PBS and centrifuging, each well was divided equally into three flow tubes, one of which remained unstained. The other two tubes were stained for 30 min in the dark at room temperature with one of two combinations. The first combination was CD86 antibody-FITC and CD64 antibody-PerCP-Cy5.5, and the second was CD11b antibody-FITC and CD206 antibody-PE, all from (Thermofisher, USA). 100 μL of staining buffer (BD Biosciences) and 5 μL from each antibody were added to every single tube. Followed by a washing step and resuspended in 200 μL of PBS. The tubes were read on FACSDiva 8 software and FACSCanto II (BD Biosciences, USA)

### 2.5 Fluorescence imaging and analysis

To study the membrane order of M0, M1, and M2 macrophages, cells were seeded in 12 well plates; the seeding density was 0.3 × 10^6^ cells/well. The cells were differentiated and polarized to M0 and M1, respectively, followed by staining the cells with 2:1000 Di-4-ANEPPDHQ: free serum media for 1 h in the incubator. Then, the cells were fixed with 0.5 mL of 4 % formaldehyde for 20 min at room temperature in the dark. A washing step with PBS followed that, and then 0.5 mL of ammonium chloride was added for 10 min in the dark at room temperature. A washed step was then done. After that, 0.5 mL of 1:1500 DAPI: PBS was added to the cells for 15 min and incubated in the dark at room temperature. Finally, the cells were washed once with 0.5 mL of PBS, and the coverslips were placed face down on a glass slide by putting a drop of mounting medium (Dako, USA), then were left to dry overnight in the dark.

Stained cells were imaged using a Zeiss LSM780 confocal microscope system (Carl Zeiss AG, Germany); a 488 nm diode laser was used to excite the Di-4-ANEPPDHQ stained, and two intensity channels were recorded: 500−580 and 620−750 nm to collect the fluorescence. The mean fluorescence intensity was measured in the manually selected plasma membrane region of individual macrophages in ImageJ using the GP (generalized polarization analysis) plugin (http://www.optinav.com/Generalized_Polarization_Analysis.htm). The membrane regions were delineated using the freehand selection tool based on Di-4-ANEPPDHQ signal distribution, and GP values were calculated using the generalized polarization plugin with a measured calibration factor (G). The calibration factor (G) was determined using Di-4-ANEPPDHQ-stained large unilamellar vesicles (LUVs) made from defined lipid compositions to model ordered (sphingomyelin/cholesterol) and disordered (DOPC) membranes, following the method described by Owen et al. [22]. The intensities in the 580 nm (I_ordered) and 620 nm (I_disordered) channels were recorded, and G was calculated using equation G = (I_ordered - I_disordered)/(I_ordered + I_disordered), ensuring accurate GP value normalization across experimental conditions. To determine the general polarization (GP) value, we analyzed the pictures acquired and calculated the general polarizations using the equation [23]:

$$GP=\frac{I_{500-580}-{GI}_{620-750}}{I_{500-580}+{GI}_{620-750}}$$

Where G is the calibration factor calculated using

$$G=\frac{{GP}_{ref}+{GP}_{ref}{GP}_{mes}-{GP}_{mes}-1}{{GP}_{mes}+{GP}_{ref}{GP}_{mes}-{GP}_{ref}-1}$$

and the ratio of M1/M2 fluorescence intensity was calculated to determine the phenotype classification.

### 2.6 Statistical analysis

The analyses were performed using GraphPad Prism version 8, ensuring a thorough statistical evaluation of the results. An unpaired two-tailed *t*-test compared means among M0, M1, and M2 groups, with a significance threshold set at p < 0.05. Effect sizes were calculated using R-squared (η^2^) statistics to evaluate the practical significance of the findings. An F-test was conducted to compare variances, confirming the assumption of equal variances for t-tests. Additionally, gene expression levels from RT-qPCR were quantified using the 2^^−ΔΔCq^ method.

## 3 Results

### 3.1 RT-qPCR analysis of cytokine expression

The RT-qPCR analysis was performed to compare the expression levels of cytokines (IL-10, IL-1β, and IL-6) across different macrophage phenotypes (M0, M1, and M2). The results are presented below.

#### 3.1.1 IL-1β expression

**Fig. 1:**
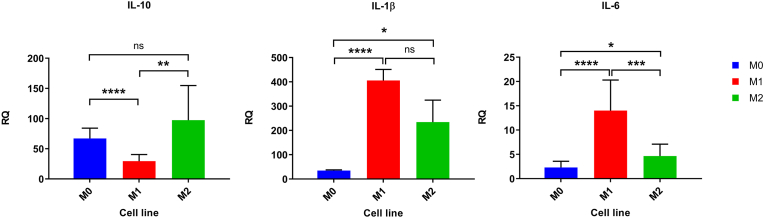
Relative quantification (RQ) of IL-1β, IL-6, and IL-10 expression in M0, M1, and M2 macrophages. M0 (Resting-state), M1 (pro-inflammatory), and M2 (anti-inflammatory) macrophages are represented by blue, red, and green bars, respectively. Statistical significance is indicated by *p*-values (*p* < 0.05 ∗, *p* < 0.01 ∗∗, *p* < 0.001 ∗∗∗, *p* < 0.0001 ∗∗∗∗, and ns for non-significant). Data represents the mean ± SEM from three independent biological replicates (n = 3). Statistical significance was determined using unpaired two-tailed t-tests.

IL-1β expression between M1 and M2 macrophages showed a non-significant mean difference of −171.3 ± 101.0 (95 % CI: −385.3 to 42.79; t = 1.69, df = 16, p = 0.11). The effect size was moderate (R^2^ = 0.15), and the F-test for variance comparison yielded a p-value of 0.07. While M0 and M2 macrophages revealed a significant mean difference of 199.6 ± 90.23 (95 % CI: 8.37 to 390.9; t = 2.21, df = 16, p = 0.021). The effect size was moderate (R^2^ = 0.23), and the F-test for variance comparison yielded a p-value of <0.0001. On the other side, a significant mean difference of 370.9 ± 45.53 (95 % CI: 274.4 to 467.4; t = 8.147, df = 16, p < 0.0001) were shown between M0 and M1 macrophages. The effect size was exceptionally large (R^2^ = 0.81), and the F-test for variance comparison yielded a p-value of <0.0001 (Supplementary Table 1) and (Fig. 1).

#### 3.1.2 IL-6 expression

Supplementary Table 2 which shows the IL-6 expression between M1 and M2 macrophages revealed a significant mean difference of −9.36 ± 2.25 (95 % CI: −14.12 to −4.59; t = 4.17, df = 16, p = 0.0007). The effect size was large (R^2^ = 0.52), and the F-test for variance comparison yielded a p-value of 0.015. M0 and M2 macrophages comparison showed a significant mean difference of 2.36 ± 0.92 (95 % CI: 0.42 to 4.30; t = 2.58, df = 16, p = 0.010). The effect size was moderate (R^2^ = 0.29), and the F-test for variance comparison yielded a p-value of 0.084. In addition, M0 and M1 macrophages revealed a significant mean difference of 11.71 ± 2.14 (95 % CI: 7.18 to 16.24; t = 5.48, df = 16, p < 0.0001). The effect size was large (R^2^ = 0.65), and the F-test for variance comparison yielded a p-value of 0.0002 (Fig. 1).

#### 3.1.3 IL-10 expression

The IL-10 expression between M1 and M2 macrophages showed a significant mean difference of 67.97 ± 19.43 (95 % CI: 26.77 to 109.2; t = 3.49, df = 16, p = 0.003). The effect size (R^2^) was 0.43, indicating that the group difference explained a moderate proportion of the variance. The F-test for variance comparison yielded a p-value of 0.0001, suggesting significant differences in variance between the groups. Meanwhile, M0 and M2 macrophages displayed a non-significant mean difference of 30.28 ± 19.91 (95 % CI: −11.92 to 72.49; t = 1.521, df = 16, p = 0.074). The effect size was smaller (R^2^ = 0.13), and the F-test for variance comparison yielded a p-value of 0.002. M0 and M1 macrophages exposed a significant mean difference of −37.69 ± 6.74 (95 % CI: −51.97 to −23.40; t = 5.59, df = 16, p < 0.0001). The effect size was large (R^2^ = 0.66), and the F-test for variance comparison yielded a p-value of 0.24 (Supplementary Table 3) and (Fig. 1).

### 3.2 Flow cytometry analysis of macrophage markers

The flow cytometry analysis was conducted to compare the expression levels of various macrophage markers (CD86, CD64, and CD206) across different macrophage phenotypes (M0, M1, and M2). The results are presented below.

#### 3.2.1 CD11b expression

The CD11b expression between THP-1 and M0 macrophages, which shown in (Table 1), displayed a significant mean difference of 424.7 ± 20.84 (95 % CI: 366.8 to 482.5; t = 20.37, df = 4, p < 0.0001). The effect size was also large (R^2^ = 0.99), and the F-test for variance comparison yielded a p-value of 0.185.

**Table 1:** Statistical comparison of CD11b expression between THP-1, and M0 groups.

Parameter	THP-1 vs M0
**Mean of Group 1**	6.33 (THP-1)
**Mean of Group 2**	431 (M0)
**Mean Differenc**e ± **SEM**	424.7 ± 20.84
**95 % Confidence Interval**	366.8 to 482.5
**t-Statistic, df**	t = 20.37 df = 4
**P-Value**	<0.0001
**Effect Size (R^2^)**	0.99
**Variance Comparison (F-Test)**	P = 0.185

#### 3.2.2 CD86 expression

**Fig. 2:**
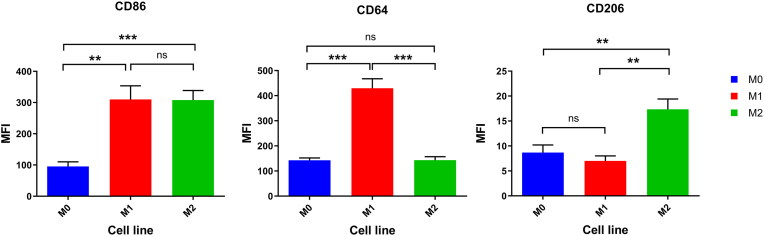
Bar graph showing the expression levels of CD86, CD64, and CD206 surface markers in M0, M1, and M2 macrophages as determined by flow cytometry. The data represent the mean ± SEM. M1 macrophages exhibited higher CD64 expression, M2 macrophages showed elevated CD206 expression, and both M1 and M2 had increased CD86 levels compared to M0.

**Fig. 3:**
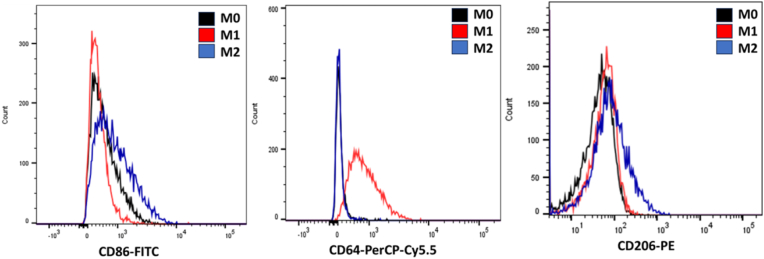
Flow cytometry histograms illustrating the fluorescence intensity distribution for CD86, CD64, and CD206 in M0, M1, and M2 macrophages. The figure highlights phenotypic differences between macrophage subsets, with distinct expression profiles supporting polarization status.

In Supplementary Table 4, the CD86 expression between M1 and M2 macrophages revealed a mean difference of −2.00 ± 31.00, with a 95 % confidence interval (CI) ranging from −88.07 to 84.07. The t-statistic was 0.065 with 4 degrees of freedom (df), and the p-value was 0.95, indicating no significant difference between the two groups. The effect size (R^2^) was 0.001, indicating minimal variance explained by the group difference. The variance comparison using the F-test yielded a p-value of 0.67, further assuring the lack of significant difference. In contrast, the expression of M0 and M2 macrophages showed a significant mean difference of 212.3 ± 19.84 (95 % CI: 157.3 to 267.4; t = 10.70, df = 4, p = 0.0004). The effect size was substantial (R^2^ = 0.97), implying that the group difference explained a considerable proportion of the variance. The F-test for variance comparison yielded a p-value of 0.37. Similarly, the comparison between M0 and M1 macrophages revealed a significant mean difference of 214.3 ± 26.72 (95 % CI: 140.1 to 288.5; t = 8.020, df = 4, p = 0.0013). The effect size was also large (R^2^ = 0.94), and the F-test for variance comparison yielded a p-value of 0.21 (Fig. 2, Fig. 3).

#### 3.2.3 CD64 expression

The analysis of CD64 expression between M1 and M2 macrophages demonstrates a significant mean difference of −286.3 ± 23.42 (95 % CI: −351.4 to −221.3; t = 12.23, df = 4, p = 0.0003). The effect size was large (R^2^ = 0.98), indicating that the group difference explained all the variance. The F-test for variance comparison yielded a p-value of 0.24. A non-significant mean difference was shown between M0 and M2 macrophages 0.33 ± 9.63 (95 % CI: −26.41 to 27.08; t = 2.213, df = 16, p = 0.97). The effect size was negligible (R^2^ = 0.0003), and the F-test for variance comparison yielded a p-value of 0.59. The comparison between M0 and M1 macrophages showed a significant mean difference of 286.7 ± 22.60 (95 % CI: 223.9 to 349.4; t = 12.69, df = 4, p = 0.0002). The effect size was exceptionally large (R^2^ = 0.98), and the F-test for variance comparison yielded a p-value of 0.11 (Supplementary Table 5) and (Fig. 2, Fig. 3).

#### 3.2.4 CD206 expression

CD206 expression between M1 and M2 macrophages demonstrate a significant mean of difference of 10.33 ± 1.33 (95 % CI: 6.63 to 14.04; t = 7.75, df = 4, p = 0.0015). The effect size was large (R^2^ = 0.94), and the F-test for variance comparison yielded a p-value of 0.38. A significant mean difference of 8.67 ± 1.49 (95 % CI: 4.53 to 12.8; t = 5.81, df = 4, p = 0.004) was disclosed between M0 and M2 macrophages. The effect size was substantial (R^2^ = 0.89), and the F-test for variance comparison yielded a p-value of 0.70. In contrast, M0 and M1 macrophages revealed a non-significant mean difference of −1.67 ± 1.05 (95 % CI: −4.59 to 1.26; t = 1.58, df = 4, p = 0.19). The effect size was moderate (R^2^ = 0.39), and the F-test for variance comparison yielded a p-value of 0.60 (Supplementary Table 6) and (Fig. 2, Fig. 3).

### 3.3 Generalized polarization results

**Fig. 4:**
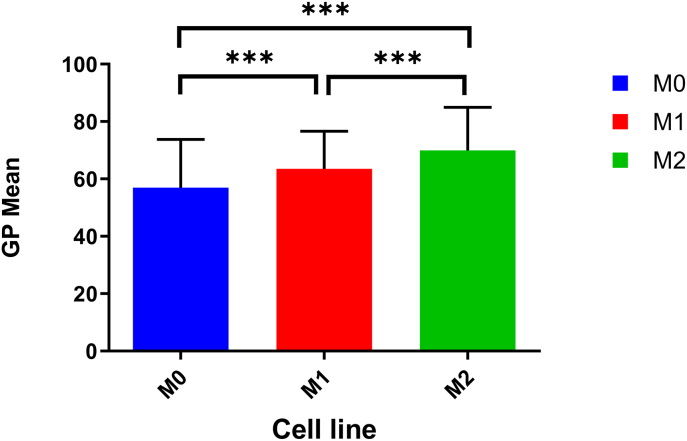
GP values were calculated for M0, M1, and M2 macrophages stained with Di-4-ANEPPDHQ. Data represent mean ± SD. Significant differences between groups are indicated by asterisks (∗∗∗p < 0.001), demonstrating notable variations in GP mean across the different macrophage phenotypes. Sample sizes: M0 (n = 36), M1 (n = 36), M2 (n = 245).

**Fig. 5:**
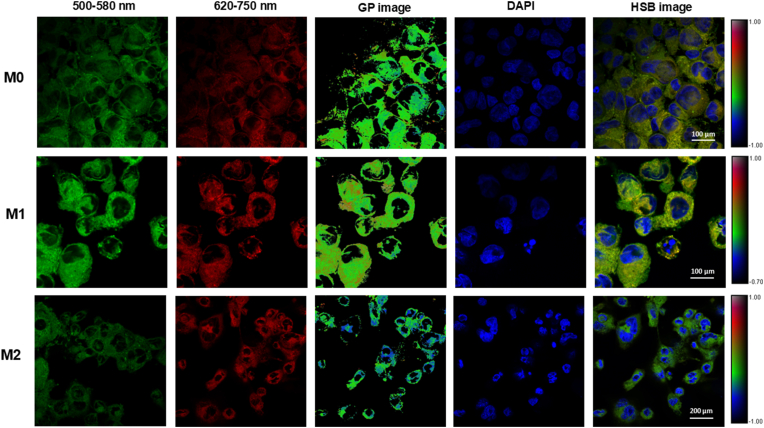
Fluorescence microscopy images of M0, M1, and M2 macrophages stained with Di-4-ANEPPDHQ (membrane order probe) and DAPI (nuclear counterstain). For each macrophage phenotype, representative images are shown from the green channel (500–580 nm, indicating more ordered membrane environments), the red channel (620–750 nm, indicating disordered membrane regions), and the corresponding GP (Generalized Polarization) map. GP images were generated by calculating pixel-wise GP values and were then pseudocolored to reflect membrane order (blue = more ordered; red = more disordered). To preserve structural information, the pseudocolored GP maps were merged with mean fluorescence intensity images using hue-saturation-brightness (HSB) color space transformation. All images were acquired under identical microscope settings.

The GP values, which reflect membrane order (Fig. 5), between M0 and M1 groups revealed a statistically significant difference in their means, as assessed by an unpaired two-tailed *t*-test (t = 4.03, df = 35, *P* < 0.0001). The mean value of (M0) was 56.98, while the mean value of (M1) was 63.56, indicating an increase of 6.58 ± 1.64 (mean difference ± SEM) in the M1 group compared to M0. The 95 % confidence interval for the difference ranged from 3.36 to 9.79, further supporting the strength of the observed effect. The calculated R-squared (η^2^) value was 0.04, reflecting a small but significant effect size. Additionally, an F-test to compare variances showed a significant difference between the two groups (F = 1.66, *P* = 0.0012), suggesting unequal variances. The reduced brightness in the 500–580 nm channel for M2 macrophages reflects a blue shift in membrane order, consistent with their hyperpolarized membrane state. All images were captured under identical settings, and differences reflect true biological variance in emission profiles. Together, these findings demonstrate a significant increase in the measured parameter in the M1 group compared to M0, with small variability and reliable confidence in the results (Supplementary Table 7) and (Fig. 4).

A significant difference was observed between the M0 and M2 groups, as determined by an unpaired two-tailed *t*-test (t = 6.82, df = 279, *P* < 0.0001). The mean value of (M0) was 56.98, while the mean value of (M2) was 69.99, corresponding to an increase of 13.01 ± 1.91 (mean difference ± SEM) in the M2 group compared to M0. The 95 % confidence interval for this difference ranged from 9.25 to 16.76, providing convincing evidence for the observed effect. The calculated R-squared (η^2^) value was 0.14, indicating a moderate effect size. An F-test for variances between the two groups revealed no significant difference (*P* = 0.16), suggesting homogeneity of variances. Collectively, these results demonstrate a significant and robust increase in the measured parameter for the M2 group relative to M0, with a moderate effect size and consistent variance between the groups (Supplementary Table 7) and (Fig. 4).

The GP values between the M1 and M2 groups revealed a statistically significant difference in their means, as determined by an unpaired two-tailed *t*-test (t = 4.58, df = 40, *P* < 0.0001). The mean value of (M1) was 63.56, while the mean value of (M2) was 69.99, corresponding to an increase of 6.43 ± 1.40 (mean difference ± SEM) in the M2 group compared to M1. The 95 % confidence interval for the difference ranged from 3.67 to 9.18, providing robust evidence for the observed effect. The R-squared (η^2^) value was 0.050, indicating a small to moderate effect size. An F-test to compare variances showed no significant difference between the two groups (*P* = 0.058), suggesting equal variances. These findings highlight a significant increase in the measured parameter in the M2 group relative to M1, with small variability and moderate confidence in the effect size (Supplementary Table 7) and (Fig. 4).

To better visualize membrane order heterogeneity within individual macrophages, we generated GP images from the Di-4-ANEPPDHQ fluorescence channels. These GP maps provide a pixel-level representation of membrane fluidity, confirming that M2 macrophages exhibit higher GP values consistent with increased membrane order, while M1 macrophages display lower GP values indicating more disordered membranes. This spatial information complements the quantitative GP measurements and highlights membrane domain organization during macrophage activation (Fig. 5) and (Supplementary Fig. 1).

### 3.4 Comparison between three methods

The study employed RT-qPCR, flow cytometry, and Di-4-ANEPPDHQ fluorescence to characterize the phenotypic and functional differences between M0, M1, and M2 macrophages.

Significant differences in cytokine expression profiles among the macrophage subsets were revealed by RT-qPCR analysis. Elevated expression of pro-inflammatory cytokines IL-1β and IL-6 were shown by M1 macrophages, while M2 macrophages displayed higher expression of the anti-inflammatory cytokine IL-10.

Different surface marker expression patterns were highlighted by flow cytometry, with increased levels of CD64, a marker associated with pro-inflammatory activation, in M1 macrophages, and an elevated expression of CD206, a hallmark of anti-inflammatory activation, in M2 macrophages. Notably, both M1 and M2 macrophages showed a high expression level in CD86 compared to M0, indicating the CD86 role as a general activation marker.

Complementing these findings, Di-4-ANEPPDHQ revealed distinct changes in membrane order, with M1 macrophages displaying depolarized membranes and M2 macrophages exhibiting hyperpolarized membranes, consistent with their respective activation states. These results collectively demonstrate that RT-qPCR and flow cytometry are highly effective tools for distinguishing between M0, M1, and M2 macrophages based on their cytokine and surface marker profiles, while Di-4-ANEPPDHQ fluorescence provides valuable complementary understandings into membrane property changes associated with macrophage polarization.

### 3.5 Key observations

RT-qPCR presented as a principally sensitive and specific method for detecting cytokine expression changes, with large mean differences and effect sizes observed across comparisons. For example, IL-1β expression displayed an R^2^ value of 0.81, exposing strong explanatory power, and highly significant p-values (p < 0.0001) were consistently observed, emphasizing robust statistical power. The narrow confidence intervals further indicated high precision in the measurements (Table 2 & Supplementary Table 8).

**Table 2:** RT-qPCR, Flow Cytometry, and Di-4-ANEPPDHQ Fluorescence comparison based on key criteria such as sensitivity, specificity, ease of use, cost, time efficiency, real-time monitoring, and accuracy.

Criteria	RT-qPCR	Flow Cytometry	Di-4-ANEPPDHQ Fluorescence
**Sensitivity**	(gene expression)	(surface/intracellular markers)	(membrane potential)
**Specificity**	(specific primers/probes)	(specific antibodies)	(membrane properties)
**Ease of Use**	Technically demanding	Technically demanding	Relatively simple
**Cost**	High (reagents, equipment)	High (antibodies, equipment)	High (dye, imaging equipment)
**Time Efficiency**	Time-consuming (∼6–8 h)	Moderate (∼4–6 h)	Fast (∼2–4 h)
**Real-Time Monitoring**	No	No	Yes
**Accuracy**	High (gene expression)	High (marker expression)	Moderate (membrane properties)

Likewise, flow cytometry exhibited exceptional sensitivity in detecting surface marker expression, with markers such as CD64 showing an R^2^ value of 0.98. The highly significant p-values (p < 0.001) and narrow confidence intervals reinforced the reliability and precision of this technique (Table 2 & Supplementary Table 8).

In contrast, Di-4-ANEPPDHQ fluorescence exhibited moderate mean differences and effect sizes, such as an R^2^ value of 0.1427 for M0 vs. M2 comparisons, indicating lower sensitivity compared to RT-qPCR and flow cytometry. Nevertheless, the highly significant p-values (p < 0.0001) and narrow confidence intervals confirmed their utility in providing precise and statistically robust information on membrane properties. Together, these findings highlight the complementary strengths of the three methods in characterizing macrophage activation (Table 2 & Supplementary Table 8).

## 4 Discussion

A comprehensive analysis of macrophage phenotypes (M0, M1, and M2) is introduced in this study using three different methods: RT-qPCR, flow cytometry, and Di-4-ANEPPDHQ fluorescence microscopy. Each method contributes distinctive visions into macrophage characterization, exposing significant differences in cytokine expression, surface markers, and membrane properties associated with these macrophage states.

Increase in the expression of CD11b indicates that the cells are differentiated from monocytes to M0 macrophages [24].

The results of the RT-qPCR show an up-regulation in the expression of IL-1*β* and IL-6 in the M1 compared with the M0, which was higher than the up-regulation of these cytokines in the M2, indicating proinflammatory features. On the other hand, the IL-10 was up-regulated in the M2, indicating anti-inflammatory features [8,22,25].

Flow cytometry results confirm the RT-qPCR results. All the CD markers were elevated in M1 and M2 compared to M0, which indicates that the cells are polarized and activated. An increase in the expression of CD64 in the M1 was higher than in the M2, which is known as a marker for M1. In contrast, the expression of CD206 was higher in M2 compared to the M1, which is known as a marker for M2. The elevation in CD86 was more significant in M2 than M1, despite it being considered a marker for M1. This could be due to experimental conditions or phenotypic plasticity [8,26,27].

Although individual cytokine markers did not distinguish all macrophage phenotypes across every pairwise comparison, multivariate analysis of IL-1β, IL-6, and IL-10 expression revealed robust classification power. Principal component analysis (PCA) demonstrated clear separation between M0, M1, and M2 macrophages. A logistic regression model trained on these markers achieved 100 % classification accuracy in distinguishing phenotypes. These findings emphasize the necessity of a combined marker approach for reliable identification of macrophage states.

### 4.1 RT-qPCR: sensitivity and limitations in cytokine profiling

The remarkable sensitivity of RT-qPCR in quantifying cytokine expression, particularly IL-1β (R^2^ = 0.81) and IL-6 (R^2^ = 0.65), aligns with other studies employing these cytokines as hallmarks of M1 polarization. For instance, Murray & Wynn (2011) demonstrated that IL-1β and IL-6 are robustly upregulated in classically activated macrophages, driving pro-inflammatory responses in infections and cancer [2]. RT-PCR is considered the gold standard for quantifying cytokines, including IL-1β, IL-6, and IL-10, in macrophages due to its speed, accuracy, sensitivity, and ability to avoid contamination, enabling high-throughput analysis of immune responses [28,29]. Critically, some studies question the specificity of IL-1β and IL-6 as exclusive M1 markers. IL-6 expression has been observed in IL-4-stimulated M2 macrophages in fibrotic lung models, suggesting contextual variability [30]. Our RT-qPCR results demonstrate that individual cytokine markers (e.g., IL-1β, IL-6, IL-10) do not consistently differentiate all macrophage subtypes across pairwise comparisons. For example, IL-1β levels did not differ significantly between M1 and M2, and IL-10 did not distinguish M0 from M2. These findings underscore the limitation of relying on single-gene expression for macrophage classification. Instead, a combination of cytokine markers improves discriminatory power. Future work could apply multivariate statistical models (e.g., linear discriminant analysis, principal component analysis) or machine learning classifiers to assess the joint classification potential of these markers. This integrative approach is likely to enhance the robustness and specificity of phenotype identification in complex immune settings.

### 4.2 Flow cytometry: precision and challenges in surface marker detection

Flow cytometry excelled in detecting surface markers, particularly CD64 (R^2^ = 0.9758), a result consistent with [14], who identified CD64 as a high-specificity M1 marker across human and murine models. Recent advances, such as spectral flow cytometry, have enhanced resolution for overlapping markers (e.g., CD86 vs. CD206), as demonstrated by Ref. [15] in their analysis of macrophage subsets in tissue repair. However, dependence on antibody specificity remains a concern. In addition, a study reported cross-reactivity of commercial CD206 antibodies with CD163 in M2-like tumor-associated macrophages, leading to false-positive signals—a caveat that underscores the need for rigorous antibody validation [31]. Flow cytometry enables precise characterization of macrophages through surface marker detection, but challenges include variations in marker expression intensity, asynchronism, and lineage infidelity, necessitating careful selection of markers and gating strategies for accurate analysis [32].

Overlapping surface marker expression in macrophages is an important challenge which complicates the distinction between M1 and M2a subsets. Additionally, culture conditions significantly influence marker expression, affecting detection precision [33].

The cytokine and surface marker expression patterns observed in our RT-qPCR and flow cytometry analyses align closely with established literature on macrophage activation. IL-1β and IL-6 expression in M1 macrophages and IL-10 expression in M2 macrophages are well-documented features of these subsets [2,8,24]. Similarly, elevated CD64 in M1 and CD206 in M2 macrophages have been consistently reported as distinguishing markers [14,15]. The moderate elevation of CD86 in M2 macrophages observed here may reflect context-dependent expression patterns, a phenomenon increasingly recognized in recent studies [8,25].

Despite these innovations, the method's inability to resolve membrane dynamics or metabolic states reinforces the need for complementary techniques like fluorescence microscopy.

### 4.3 Di-4-ANEPPDHQ fluorescence: novelty and contextual limitations

By facilitating key signaling, cytokine production, and metabolic processes lipid rafts play a fundamental task in the differentiation of macrophages into M1 and M2 phenotypes. M1 macrophages, activated by pro-inflammatory signals such as lipopolysaccharides (LPS) and interferon-gamma (IFN-γ), depend on lipid rafts for effective signal transduction through enriched TLRs, which promote M1 activation and enhance the production of inflammatory cytokines l TNF-α and IL-6 [34]. On the other hand, cytokines such as IL-4 and IL-13 activate M2 macrophages utilizing lipid rafts to boost anti-inflammatory signaling via the JAK/STAT6 pathway and are characterized by their dependency on lipid metabolism for proper function. Additionally, the dynamic nature of lipid rafts allows M2 macrophages to alter their composition in response to microenvironmental signals, enabling the switch between pro-inflammatory (M1) and anti-inflammatory (M2) states as required [35].

The depolarized (red shift) and hyperpolarized (blue shift) membrane states of M1 and M2 macrophages, respectively, align with prior studies linking membrane potential to activation. Jin et al. (2006) first demonstrated Di-4-ANEPPDHQ's utility in lipid raft visualization [19]. However, the technique's moderate sensitivity (R^2^ = 0.143 for M0 vs. M2) highlights contextual limitations. Despite these challenges, the method's non-antibody-based, environment-sensitive labeling and real-time imaging capabilities make it particularly valuable for live-cell studies, although in this study we used fixed cells to standardize and compare membrane order under defined experimental conditions.

Quantifying membrane order in macrophages using fluorescence microscopy can be effectively achieved through the application of generalized polarization (GP) values derived from fluorescent probes, particularly Di-4-ANEPPDHQ. This method allows to quantify membrane fluidity and order by analyzing the fluorescence emission spectra of the probe, where a higher GP value indicates a more ordered membrane, and a lower GP value suggests increased fluidity. By utilizing Di-4-ANEPPDHQ, the biophysical properties of macrophage membranes can be revealed, especially in relation to their activation states and functional responses during processes like phagocytosis. The dye's sensitivity to membrane lipid packing enables quantification of membrane order through the Generalized Polarization (GP) metric. Our findings demonstrate that activated macrophages (e.g., LPS-stimulated) exhibit increased membrane order compared to non-activated controls, particularly at the leading edge and phagocytic cups. These changes reflect lipid raft clustering and cytoskeletal remodeling required for phagocytic activity. Conversely, anti-inflammatory stimuli (e.g., IL-4) induced distinct membrane patterns with lower GP values, consistent with alternative activation pathways. Thus, Di-4-ANEPPDHQ imaging provides a quantitative, spatially resolved readout of how membrane organization correlates with functional states of macrophages [[36], [37], [38]].

Although our study utilized confocal fluorescence microscopy to measure GP values of macrophage membranes, similar analyses can potentially be adapted for flow cytometry. With appropriate configuration—using a 488 nm excitation source and dual-emission detection (e.g., FL1 and FL3 or FL2 and FL4 channels) flow cytometry can detect Di-4-ANEPPDHQ's red and green emissions, allowing ratiometric GP calculations. This method offers the advantage of rapid, high-throughput quantification of membrane order in thousands of cells, making it suitable for population-level analysis. However, unlike fluorescence imaging, flow cytometry lacks spatial resolution, which is crucial for assessing subcellular membrane organization and local heterogeneities (e.g., at phagocytic cups or leading edges). Moreover, flow-based GP measurements require rigorous compensation and calibration to ensure spectral separation and accurate ratio calculation. Therefore, while flow cytometry may complement imaging-based GP assessment by enhancing sample size and statistical power, confocal imaging remains superior for visualizing membrane dynamics and heterogeneity within individual cells.

In addition, our findings suggest that Di-4-ANEPPDHQ is best employed in combination with conventional markers. While it does not independently resolve macrophage polarization states with the specificity of flow cytometry or qPCR, it contributes additional functional information related to membrane order and potential. Such biophysical insights can be especially valuable in live-cell imaging workflows or when characterizing transient or plastic polarization states.

### 4.4 Study limitations

Several limitations may affect the interpretation of findings of this study. It principally employs THP-1 monocyte-derived macrophages, which may not accurately represent the complexity of *in vivo* macrophages, limiting the generalizability of the results. The lack of *in vivo* validation confines the applicability of the findings to biological systems. Additionally, while RT-qPCR shows up its sensitivity, the study does not address the dynamic nature of cytokine expression over time. Concerns regarding antibody cross-reactivity in flow cytometry are not thoroughly discussed, which could lead to misinterpretation of surface marker expression. Finally, the manuscript briefly mentions variability in cytokine expression but does not explore how different stimuli might influence macrophage activation, limiting the depth of analysis.

### 4.5 Future directions

To enhance translational relevance and better resolve heterogeneity within macrophage populations, future studies will incorporate primary human monocyte-derived macrophages and adopt hybrid approaches such as coupling single-cell RNA sequencing (scRNA-seq) with fluorescence imaging [39]. These integrative frameworks will improve our understanding of macrophage plasticity and help validate whether Di-4-ANEPPDHQ can reliably distinguish M1 and M2 activation states in more physiologically relevant systems, ultimately supporting therapeutic advancements in diseases such as cancer and fibrosis. While our study demonstrates that Di-4-ANEPPDHQ can distinguish macrophage phenotypes based on membrane order in THP-1-derived cells, future studies should include primary macrophages—such as mouse bone marrow-derived macrophages (BMDMs) to validate the generalizability of this approach. BMDMs are widely used in polarization studies and exhibit more physiologically relevant phenotypes than immortalized cell lines. Assessing whether Di-4-ANEPPDHQ labeling can similarly resolve M1 and M2 phenotypes in BMDMs would provide critical insight into the robustness and translational potential of this membrane-based classification method.

To further explore the utility of Di-4-ANEPPDHQ in live-cell applications, future studies will incorporate time-lapse imaging to monitor macrophage repolarization under dynamic conditions. This will allow real-time tracking of membrane order changes in response to external stimuli and may help uncover transient intermediate states that are not readily identified by classical markers.

## CRediT authorship contribution statement

**Ahmed Abu Siniyeh:** Writing – review & editing, Writing – original draft, Validation, Supervision, Project administration, Methodology, Conceptualization. **Walhan Alshaer:** Writing – review & editing, Methodology, Data curation. **Nirmeen Elzogheir:** Methodology, Investigation, Data curation. **Majed Al-Holi:** Methodology, Investigation, Data curation. **Dana A. Alqudah:** Investigation, Data curation. **Duaa Abuarqoub:** Methodology, Investigation, Formal analysis, Data curation. **Joanna M. Kwiatek:** Writing – review & editing, Validation.

## Funding statement

This research received no specific grant from any funding agency in the public, commercial, or not-for-profit sectors.

## Declaration of competing interest

The authors declare that they have no known competing financial interests or personal relationships that could have appeared to influence the work reported in this paper.

## Data Availability

Data will be made available on request.
