# Supplementary material for: Comparative analysis of RT-qPCR, flow cytometry, and Di-4-ANEPPDHQ fluorescence for distinguishing macrophages phenotypes
Source: Biochem Biophys Rep. 2025 Aug 30;44:102225. doi: 10.1016/j.bbrep.2025.102225 (PMC12418842; doi:10.1016/j.bbrep.2025.102225)
Supplement: Multimedia component 1 [file mmc1.docx]

**Supplementary Table 1:** Statistical comparison of IL-1β expression between M1, M2, and M0 groups.

| Parameter | M1 vs M2 | M0 vs M2 | M0 vs M1 |
| --- | --- | --- | --- |
| Mean of Group 1 | 405.7 (M1) | 34.82 (M0) | 34.82 (M0) |
| Mean of Group 2 | 234.5 (M2) | 234.5 (M2) | 405.7 (M1) |
| Mean Difference ± SEM | -171.3 ± 101.0 | 199.6 ± 90.23 | 370.9 ± 45.53 |
| 95% Confidence Interval | -385.3 to 42.79 | 8.365 to 390.9 | 274.4 to 467.4 |
| t-Statistic, df | t=1.69 df=16 | t=2.21 df=16 | t=8.15 df=16 |
| P-Value | 0.11 | 0.021 | < 0.0001 |
| Effect Size (R²) | 0.15 | 0.23 | 0.81 |
| Variance Comparison (F-Test) | P = 0.07 | P < 0.0001 | P < 0.0001 |

**IL-6 Expression**

Table 2 which shows the IL-6 expression between M1 and M2 macrophages revealed a significant mean difference of -9.36 ± 2.25 (95% CI: -14.12 to -4.59; t=4.17, df=16, p=0.0007).

**Supplementary Table 2**: Statistical comparison of IL-6 expression between M1, M2, and M0 groups.

| Parameter | M1 vs M2 | M0 vs M2 | M0 vs M1 |
| --- | --- | --- | --- |
| Mean of Group 1 | 14.02 (M1) | 2.30 (M0) | 2.30 (M0) |
| Mean of Group 2 | 4.66 (M2) | 4.66 (M2) | 14.02 (M1) |
| Mean Difference ± SEM | -9.36 ± 2.25 | 2.357 ± 0.92 | 11.71 ± 2.14 |
| 95% Confidence Interval | -14.12 to -4.59 | 0.4174 to 4.30 | 7.183 to 16.24 |
| t-Statistic, df | t=4.17 df=16 | t=2.58 df=16 | t=5.48 df=16 |
| P-Value | 0.0007 | 0.010 | < 0.0001 |
| Effect Size (R²) | 0.52 | 0.29 | 0.65 |
| Variance Comparison (F-Test) | P = 0.015 | P = 0.084 | P = 0.0002 |

**Supplementary Table 3**: Statistical comparison of IL-10 expression between M1, M2, and M0 groups.

| Parameter | M1 vs M2 | M0 vs M2 | M0 vs M1 |
| --- | --- | --- | --- |
| Mean of Group 1 | 29.46 (M1) | 67.15 (M0) | 67.15 (M0) |
| Mean of Group 2 | 97.43 (M2) | 97.43 (M2) | 29.46 (M1) |
| Mean Difference ± SEM | 67.97 ± 19.43 | 30.28 ± 19.91 | -37.69 ± 6.74 |
| 95% CI | 26.77 to 109.2 | -11.92 to 72.49 | -51.97 to -23.40 |
| t-Statistic, df | t=3.498 df=16 | t=1.521 df=16 | t=5.593 df=16 |
| P-Value | 0.003 | 0.074 | < 0.0001 |
| Effect Size (R²) | 0.43 | 0.13 | 0.66 |
| Variance Comparison (F-Test) | P = 0.0001 | P = 0.0024 | P = 0.24 |

**Supplementary Table 4:** Statistical comparison of CD86 expression between M1, M2, and M0 groups.

| Parameter | M1 vs M2 | M0 vs M2 | M0 vs M1 |
| --- | --- | --- | --- |
| Mean of Group 1 | 309.7 (M1) | 95.33 (M0) | 95.33 (M0) |
| Mean of Group 2 | 307.7 (M2) | 307.7 (M2) | 309.7 (M1) |
| Mean Difference ± SEM | -2.00 ± 31.00 | 212.3 ± 19.84 | 214.3 ± 26.72 |
| 95% Confidence Interval | -88.07 to 84.07 | 157.3 to 267.4 | 140.1 to 288.5 |
| t-Statistic, df | t=0.065 df=4 | t=10.70 df=4 | t=8.020 df=4 |
| P-Value | 0.95 | 0.0004 | 0.0013 |
| Effect Size (R²) | 0.001 | 0.97 | 0.94 |
| Variance Comparison (F-Test) | P = 0.67 | P = 0.37 | P = 0.21 |

**CD64 Expression**

The analysis of CD64 expression between M1 and M2 macrophages showed a significant mean difference of -286.3 ± 23.42 (95% CI: -351.4 to -221.3; t=12.23, df=4, p=0.0003). The effect size

**Supplementary Table 5:** Statistical comparison of CD64 expression between M1, M2, and M0 groups.

| Parameter | M1 vs M2 | M0 vs M2 | M0 vs M1 |
| --- | --- | --- | --- |
| Mean of Group 1 | 429.3 (M1) | 142.7 (M0) | 142.7 (M0) |
| Mean of Group 2 | 143 (M2) | 143 (M2) | 429.3 (M1) |
| Mean Difference ± SEM | -286.3 ± 23.42 | 0.33 ± 9.63 | 286.7 ± 22.60 |
| 95% Confidence Interval | -351.4 to -221.3 | -26.41 to 27.08 | 223.9 to 349.4 |
| t-Statistic, df | t=12.23 df=4 | t=2.213 df=16 | t=12.69 df=4 |
| P-Value | 0.0003 | 0.97 | 0.0002 |
| Effect Size (R²) | 0.98 | 0.0003 | 0.98 |
| Variance Comparison (F-Test) | P = 0.24 | P = 0.59 | P = 0.11 |

**Supplementary Table 6:** Statistical comparison of CD206 expression between M1, M2, and M0 groups.

| Parameter | M1 vs M2 | M0 vs M2 | M0 vs M1 |
| --- | --- | --- | --- |
| Mean of Group 1 | 7 (M1) | 8.67 (M0) | 8.67 (M0) |
| Mean of Group 2 | 17.33 (M2) | 17.33 (M2) | 7 (M1) |
| Mean Difference ± SEM | 10.33 ± 1.33 | 8.67 ± 1.49 | -1.67 ± 1.05 |
| 95% Confidence Interval | 6.63 to 14.04 | 4.53 to 12.8 | -4.59 to 1.26 |
| t-Statistic, df | t=7.75 df=4 | t=5.81 df=4 | t=1.58 df=4 |
| P-Value | 0.0015 | 0.004 | 0.19 |
| Effect Size (R²) | 0.94 | 0.89 | 0.39 |
| Variance Comparison (F-Test) | P = 0.38 | P = 0.70 | P = 0.60 |

**Supplementary Table 7:** statistical comparisons of GP values among groups M1, M0, and M2.

| Parameter | M1 vs M2 | M0 vs M2 | M0 vs M1 |
| --- | --- | --- | --- |
| Mean of Group 1 | 63.56 (M1) | 56.98 (M0) | 56.98 (M0) |
| Mean of Group 2 | 69.99 (M2) | 69.99 (M2) | 63.56 (M1) |
| Mean Difference ± SEM | 6.43 ± 1.40 | 13.01 ± 1.91 | 6.58 ± 1.64 |
| 95% Confidence Interval | 3.67 to 9.18 | 9.25 to 16.76 | 3.36 to 9.79 |
| t-Statistic, df | t = 4.58, df = 401 | t = 6.82, df = 279 | t = 4.03, df = 350 |
| P-Value | < 0.0001 | < 0.0001 | < 0.0001 |
| Effect Size (R²) | 0.050 | 0.143 | 0.044 |
| Variance Comparison (F-Test) | P = 0.058 | P = 0.16 | P = 0.0012 |

**Supplementary Table 8:** This table presents a comparative analysis of RT-qPCR, Flow Cytometry, and Di-4-ANEPPDHQ Fluorescence in assessing IL-1β, IL-6, IL-10, CD86, CD64, CD206, and membrane order (GP). Significant differences (p < 0.05) indicate distinct expression patterns among macrophage subsets**.**

| Method | Comparison | Mean Difference ± SEM | 95% CI | p-value | Effect Size (R²) |
| --- | --- | --- | --- | --- | --- |
| RT-qPCR (IL-1β) | M0 vs. M1 | 370.9 ± 45.53 | 274.4 to 467.4 | < 0.0001 | 0.81 |
|  | M0 vs. M2 | 199.6 ± 90.23 | 8.37 to 390.9 | 0.021 | 0.23 |
|  | M1 vs. M2 | -171.3 ± 101.0 | -385.3 to 42.79 | 0.11 | 0.15 |
| RT-qPCR (IL-6) | M0 vs. M1 | 11.71 ± 2.14 | 7.18 to 16.24 | < 0.0001 | 0.65 |
|  | M0 vs. M2 | 2.36 ± 0.92 | 0.42 to 4.29 | 0.010 | 0.29 |
|  | M1 vs. M2 | -9.356 ± 2.25 | -14.12 to -4.59 | 0.0007 | 0.52 |
| RT-qPCR (IL-10) | M0 vs. M1 | -37.69 ± 6.74 | -51.97 to -23.40 | < 0.0001 | 0.66 |
|  | M0 vs. M2 | 30.28 ± 19.91 | -11.92 to 72.49 | 0.074 | 0.13 |
|  | M1 vs. M2 | 67.97 ± 19.43 | 26.77 to 109.2 | 0.0030 | 0.43 |
| Flow Cytometry (CD86) | M0 vs. M1 | 214.3 ± 26.72 | 140.1 to 288.5 | 0.0013 | 0.94 |
|  | M0 vs. M2 | 212.3 ± 19.84 | 157.3 to 267.4 | 0.0004 | 0.97 |
|  | M1 vs. M2 | -2.00 ± 31.00 | -88.07 to 84.07 | 0.95 | 0.001 |
| Flow Cytometry (CD64) | M0 vs. M1 | 286.7 ± 22.60 | 223.9 to 349.4 | 0.0002 | 0.98 |
|  | M0 vs. M2 | 0.33 ± 9.63 | -26.41 to 27.08 | 0.97 | 0.0003 |
|  | M1 vs. M2 | -286.3 ± 23.42 | -351.4 to -221.3 | 0.0003 | 0.97 |
| Flow Cytometry (CD206) | M0 vs. M1 | -1.67 ± 1.054 | -4.59 to 1.26 | 0.19 | 0.39 |
|  | M0 vs. M2 | 8.67 ± 1.49 | 4.53 to 12.8 | 0.004 | 0.89 |
|  | M1 vs. M2 | 10.33 ± 1.33 | 6.63 to 14.04 | 0.002 | 0.94 |
| Di-4-ANEPPDHQ  (GP) | M0 vs. M1 | 6.58 ± 1.64 | 3.36 to 9.79 | < 0.0001 | 0.04 |
|  | M0 vs. M2 | 13.01 ± 1.91 | 9.25 to 16.76 | < 0.0001 | 0.14 |
|  | M1 vs. M2 | 6.43 ± 1.40 | 3.67 to 9.18 | < 0.0001 | 0.05 |

**
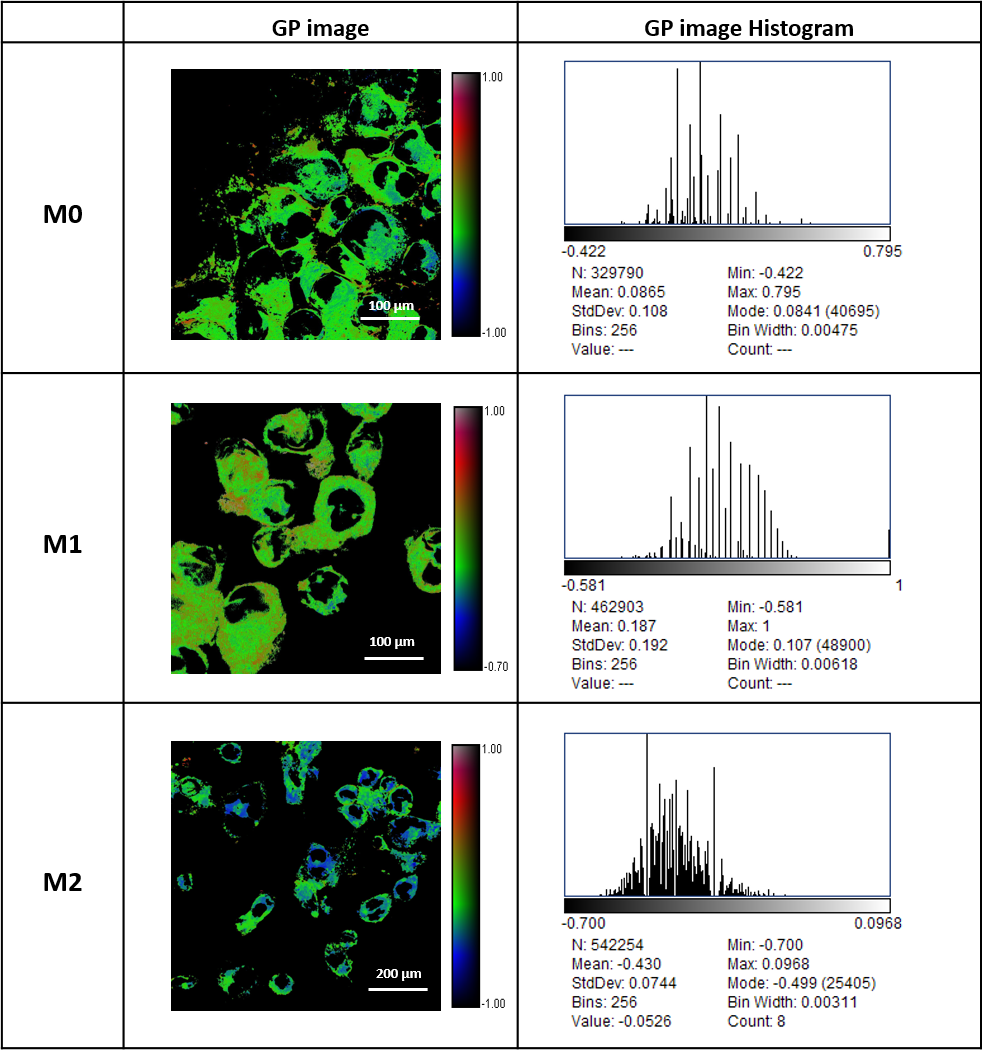
**

**Supplementary Figure 1:** Histograms of generalized polarization (GP) values for M0, M1, and M2 macrophages. Pixel-wise GP values were calculated from Di-4-ANEPPDHQ fluorescence images, and their frequency distributions were plotted across the full GP range (–1 to +1). Each histogram represents the distribution of membrane order within a single representative field for each macrophage phenotype. Higher GP values indicate more ordered membranes, while lower GP values correspond to more disordered membranes. These histograms complement the pseudocolored GP maps in Figure 5 and provide a quantitative comparison of membrane biophysical properties.
