# Supplementary material for: Comparative analysis of RT-qPCR, flow cytometry, and Di-4-ANEPPDHQ fluorescence for distinguishing macrophages phenotypes
Source: Biochem Biophys Rep. 2025 Aug 30;44:102225. doi: 10.1016/j.bbrep.2025.102225 (PMC12418842; doi:10.1016/j.bbrep.2025.102225)
Supplement: Multimedia component 2 [file mmc2.pdf]

ResearchGate

See discussions, stats, and author profiles for this publication at: https://www.researchgate.net/publication/51868025

Quantitative imaging of membrane lipid order in cells and organisms

Article *in* Nature Protocol · December 2011

DOI: 10.1038/nprot.2011.419 · Source: PubMed

CITATIONS
291

READS
1,919

5 authors, including:

Carles Rentero
University of Barcelona
92 PUBLICATIONS **2,795** CITATIONS
SEE PROFILE

Astrid Magenau
Garvan Institute for Medial Research
82 PUBLICATIONS **2,402** CITATIONS
SEE PROFILE

Ahmed Abu siniyeh
University of Jordan
16 PUBLICATIONS **391** CITATIONS
SEE PROFILE

Some of the authors of this publication are also working on these related projects:

Project

Role of LDL cholesterol, SRC and annexin A6 for integrin dependent cancer cell migration View project

All content following this page was uploaded by Ahmed Abu siniyeh on 23 May 2014.

The user has requested enhancement of the downloaded file.

Quantitative imaging of membrane lipid order in cells and organisms

Dylan M Owen[1], Carles Rentero[2], Astrid Magenau[1], Ahmed Abu-Siniyeh[1] & Katharina Gaus[1]

[1]Centre for Vascular Research, University of New South Wales, Sydney, Australia. [2]Facultat de Medicina, Departament de Biologia Cel·lular, Immunologia i Neurociències, Institut d'Investigacions Biomèdiques August Pi i Sunyer (IDIBAPS), Universitat de Barcelona, Barcelona, Spain. Correspondence should be addressed to D.M.O. (dylan.owen@unsw.edu.au) or K.G. (k.gaus@unsw.edu.au).

Published online 8 December 2011; doi:10.1038/nprot.2011.419

It is now recognized that lipids and proteins in cellular membranes are not homogenously distributed. A high degree of membrane order is the biophysical hallmark of cholesterol-enriched lipid rafts, which may induce the lateral sorting of proteins within the membrane. Here we describe a quantitative fluorescence microscopy technique for imaging localized lipid environments and measuring membrane lipid order in live and fixed cells, as well as in intact tissues. The method is based on the spectral ratiometric imaging of the polarity-sensitive membrane dyes Laurdan and di-4-ANEPPDHQ. Laurdan typically requires multiphoton excitation, making it suitable for the imaging of tissues such as whole, living zebrafish embryos, whereas di-4-ANEPPDHQ imaging can be achieved with standard confocal microscopes. This approach, which takes around 4 h, directly examines the organization of cellular membranes and is distinct from alternative approaches that infer membrane order by measuring probe partitioning or dynamics.

© 2011 Nature America, Inc. All rights reserved.

INTRODUCTION

Background

In recent years, the lipid raft hypothesis has changed how cell biologists view lipids and their contribution to membrane organization. Originally formulated in 1997 (ref. 1), it created excitement because it elevated lipids from simple building blocks to regulatory elements. However, imaging lipid rafts directly in intact cells is not trivial, particularly as they are now viewed as "small (10–200 nm), heterogeneous, highly dynamic, sterol- and sphingolipid-enriched domains that compartmentalize cellular processes"[2]. Given the resolution limit of conventional optical techniques, it is therefore not possible to view individual raft domains.

Here we focus on the quantification of one of the key biophysical parameters that gives rise to lipid domains: membrane order. A highly ordered membrane environment influences the dynamics of membrane proteins[3], which in turn affects the likelihood of protein-protein interactions and hence the efficiency of signaling pathways[4]. An example in which membrane order has been linked to a cellular function is the T cell immunological synapse. High membrane order was observed at T cell receptor activation sites and the periphery of the immunological synapse[5,6]. Consequently, disrupting membrane order mislocalizes membrane proteins and impairs T cell activation[7]. Other examples in which membrane order is thought to have a role include B cell signaling[8], cell adhesion[9], viral entry/budding[10–12] and membrane trafficking[13,14]. Membrane order can be described by the degree of lipid packing, the thickness of the lipid bilayer and the rotational freedom of lipids within the bilayer. Probing the degree of lipid packing provides an opportunity to visualize membrane domains, and the degree of lipid packing can be quantified because more efficient packing excludes polar water molecules from the otherwise nonpolar bilayer, resulting in a change in local environmental polarity that can be sensed with polarity-sensitive fluorescent probes.

Weber and Farris[15] developed a series of polarity-sensitive membrane probes, of which Laurdan (6-lauryl-2-dimethylamino-napthalene) is now most commonly used (**Fig. 1a**). Similarly to most polarity-sensitive probes, Laurdan is solvatochromic and shows an increase in charge separation when excited in polar solvents, which results in a larger dipole moment. It therefore has more than one excited state: a locally excited state intrinsic to the fluorophore and an internal charge transfer state created by the larger dipole moment. These transitions from locally excited state states in nonpolar solvents to internal charge transfer states in polar solvents shift the emission maxima. In Laurdan's case, the less-polar membrane environment of the liquid-ordered phase (often regarded as synonymous with lipid rafts) induces a 50-nm blue shift in the emission maxima[16–18]. This shift in emission profile between liquid-disordered and liquid-ordered phases[16,19] (**Fig. 1c**) allows a quantitative assessment of membrane order by calculating a ratiometric measurement of the fluorescence intensity recorded in two spectral channels, known as a generalized polarization (GP) value[19,20].

Characterization of Laurdan as a means to quantify membrane order in artificial membrane systems was performed in the 1990s, mainly in the lab of Enrico Gratton[16,19,20]. Laurdan is excited at ~400 nm, making it particularly well suited for multiphoton microscopy (at ~800 nm) and therefore tissue imaging[21]. Imaging was translated into live cells by the use of multiphoton excitation using the protocol presented here[22,23], and since then Laurdan has been the most widely used dye for studying membrane order in both live- and fixed-cell systems and artificial membranes[24–27]. However, the equipment required for multiphoton excitation is relatively complex and expensive. Using the described protocol, the dye was successfully applied to imaging live zebrafish embryos by our group, the first time membrane order imaging had been achieved in whole, living vertebrate organisms[21].

Recently, a new membrane probe, di-4-ANEPPDHQ (**Fig. 1b**), has been developed[28], which works similarly to Laurdan. Di-4-ANEPPDHQ was originally developed as a probe of membrane potential[28] but was demonstrated as a probe of membrane order in artificial membranes and cells in 2005 and 2006 (refs. 29, 30). Di-4-ANEPPDHQ excites at 488 nm and can therefore be used on most standard confocal and multichannel total internal reflection fluorescence (TIRF) systems[6,30] with Argon ion lasers as an excitation source. Similarly to Laurdan, di-4-ANEPPDHQ has a 60-nm spectral blue shift between the disordered and ordered bilayer phases[30] (**Fig. 1d**), and the method previously established for Laurdan has been expanded to apply to di-4-ANEPPDHQ to allow

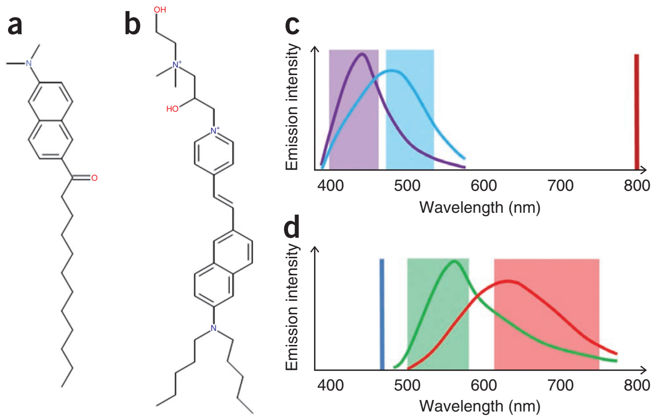

Figure 1 | Schematic of the probes and their emission spectra in different lipid environments. (**a**) Structure of Laurdan. (**b**) Structure of di-4-ANEPPDHQ. (**c**) Scheme of the fluorescence properties of Laurdan. The dye is excited using multiphoton illumination at 800 nm (red line). The dye fluoresces with a peak emission wavelength around 450 nm (violet) when residing in the ordered phase and ~500 nm in the disordered phase (blue). Two-channel acquisition is conducted in the wavelength bands indicated by shaded boxes. (**d**) Scheme of the fluorescence properties of di-4-ANEPPDHQ. The dye is excited using single-photon illumination at 488 nm (blue line). The dye fluoresces with a peak emission wavelength of ~560 nm (green) when residing in the ordered phase, and ~620 nm in the disordered phase (red). Two-channel acquisition is conducted in the wavelength bands indicated by shaded boxes.

live-cell imaging in 3D and two-channel TIRF imaging[6]. Notably, Laurdan and di-4-ANEPPDHQ have been shown to be specific probes of lipid packing, and they are not influenced by the presence of membrane-associated peptides[31].

Alternative approaches

Intensity-based measurements can be used to infer the relative distribution of protein markers, either with fluorescent fusion constructs or by immunofluorescence. Common proteins that are taken to have ordered phase or raft affinity include most glycophosphatidylinositol-anchored proteins, as well as palmitoylated proteins such as Lck or H-Ras. Most transmembrane proteins such as the phosphatase CD45 or the transferrin receptor are believed to have affinity for the disordered phase[32] and are therefore non-raft markers. Although they are simple to implement, intensity-based measurements are often imprecise, as they do not take into account the amount of membrane in any pixel; accordingly, membrane ruffling, for example, could lead to erroneous measurements. One common example of this method is the use of cholera toxin B-subunit (CTxB). CTxB binds to the lipid ganglioside G_{M1}, which is enriched in highly ordered domains. It is common to detect fluorescently tagged CTxB and infer the presence of ordered membranes from enrichment in CTxB staining. Although it is widely used, this technique has a serious drawback in that CTxB is a homo-pentamer and can bind up to five G_{M1} molecules simultaneously and therefore potentially cross-link small domains into larger structures[33].

More sophisticated imaging approaches such as single-particle tracking and fluorescence correlation spectroscopy can also be used to quantify the diffusion properties of lipids and proteins from which the existence of lipid domains can be deduced. Recently, stimulated emission depletion and fluorescence correlation spectroscopy have been combined to detect subdiffraction-sized lipid domains in cell membranes[34]. These technologies often require highly specialized equipment and do not directly measure lipid clustering or lipid domains; however, they do have the advantage of being highly sensitive single-molecule techniques that provide information on confinement zones and the cortical actin meshwork[35–38].

There are also different modalities available for the imaging of di-4-ANEPPDHQ or Laurdan. A particularly suitable approach is the use of TIRF microscopy to study the basal plasma membrane of adherent cells. For two-channel TIRF, an image splitter can be used to direct the two acquisition channels onto two different charge-coupled device cameras or to two halves of the same camera sensor. TIRF has been demonstrated previously with both di-4-ANEPPDHQ[6] and Laurdan[39] (excited with a laser diode at ~405 nm). Here Laurdan must be excited with single-photon excitation, which can cause problems of phototoxicity when performing live-cell imaging. Similarly, both dyes can be used with conventional epifluorescence imaging, albeit with a substantial increase in background. Spinning-disk confocal microscopy can also be used, as can fluorescence-activated cell sorting to sort cells based on the degree of total membrane order[40,41].

Limitations

The main limitations of the technique stem from the resolution limit of the microscope. Depending on the dye, excitation wavelength and the objective used, this will be of the order of 200–300 nm. As plasma membrane microdomains are postulated to be of the order of tens of nanometers, it is important to emphasize that the method described here cannot image individual domains. Instead, any resolution element (pixel) in the image will contain fluorescence signal originating from both disordered and ordered membrane regions. The GP value therefore gives an average measure of the membrane order in each diffraction-limited volume. The same argument applies to the time required to acquire a single image, which is typically in the order of a few seconds. If the domains are dynamic and short-lived, the GP value in any pixel will be the temporal average of the microdomain fraction over that time.

The other major limitation is the simultaneous imaging of membrane order and the localization of specific proteins and lipids. This is difficult because of the broad emission spectrum of di-4-ANEPPDHQ and the need to switch between multiphoton and confocal imaging modes with Laurdan.

Experimental design

Probe design. Although Laurdan and di-4-ANEPPDHQ have become the *de facto* standards for spectral ratiometric imaging, it should be noted that other polarity-sensitive probes have existed for some time, such as Prodan and di-8-ANEPPS, and new probes continue to be developed. These include the more sensitive Laurdan derivatives C-Laurdan[42] and C-Laurdan2 (ref. 43). The latter is a ‘turn on’ probe that only emits fluorescence when in the ordered phase. These dyes are not currently commercially available. An optimal probe for sensing membrane order should have low background fluorescence in the polar, aqueous environment and a large difference in Stokes shift between the ordered and disordered phases to achieve maximum change in emission spectra per incremental change in membrane order. Although brighter, more photostable probes are of course desirable, a second important issue is the internalization of dyes that stain intracellular membranes. Often one solely wants to image the cell plasma membrane or a particular

© 2011 Nature America, Inc. All rights reserved.

organelle; however, the rapid distribution of the probe between cellular membranes can cause difficulties in image analysis and interpretation. Finally, ideal dyes for the plasma membrane would partition approximately equally between ordered and disordered domains; they would not alter the membrane phase behavior[44] and they would have low cellular toxicity.

Choice of fluorophore. The choice of whether to use Laurdan or di-4-ANEPPDHQ depends on the biological system under investigation and the type of data required. First, Laurdan is the more suitable dye when imaging whole organisms (for example, zebrafish embryos) or thick tissue samples. Such samples are more suited to multiphoton excitation at 800 nm, at which the degree of scattering in tissue is reduced.

In cells, the choice is typically made by what equipment is available. Many confocal systems do not have either a 405-nm laser or a pulsed laser capable of multiphoton excitation for Laurdan. Almost all confocal systems, however, have a 488-nm laser (either an Argon ion or a diode-pumped solid-state laser (DPSSL)), and are therefore capable of efficiently exciting di-4-ANEPPDHQ. Di-4-ANEPPDHQ also displays lower background signal in the cytosol, which makes it easier to discern the plasma membrane and other intracellular membrane structures. Di-4-ANEPPDHQ images should have slightly higher spatial resolution because of the shorter excitation wavelengths used. Also note that both probes are applicable to imaging artificial membrane systems.

The major disadvantage of di-4-ANEPPDHQ is that its emission spectrum spans 500–750 nm and therefore covers almost the whole spectral region that is accessible to most microscope systems. This makes di-4-ANEPPDHQ difficult to multiplex with other fluorophores if simultaneous imaging is required. In contrast, Laurdan fluorescence effectively ceases above 550 nm, and it can therefore be used to simultaneously image with fluorescent protein constructs such as mCherry or small-molecule dyes such as Cy3 or Cy5. Note that it is possible to simultaneously image di-4-ANEPPDHQ and CFP; however, this requires the 'ordered phase' detection channel of di-4-ANEPPDHQ to be moved to longer wavelengths to avoid CFP bleed-through. This markedly reduces GP image contrast. It is also possible to use di-4-ANEPPDHQ with mCherry, provided that the fluorophores are imaged sequentially by exciting di-4-ANEPPDHQ at 458 nm, at which mCherry does not absorb. Conversely, di-4-ANEPPDHQ is not excited when 561 nm is used to excite mCherry. If pulsed lasers are available, this affords the opportunity to image di-4-ANEPPDHQ and mCherry simultaneously using pulsed interleaved excitation. Simultaneous Laurdan imaging with a red fluorophore will typically be done with a fully open confocal pinhole, thus reducing optical sectioning for the red fluorophore. Hence, sequential acquisition also is recommended for Laurdan.

Laurdan has an advantage when performing 3D imaging because multiphoton excitation will only bleach fluorophores in the plane currently being imaged (however, photodamage outside the focal plane can still occur). Conversely, di-4-ANEPPDHQ is in general more photostable and therefore is more suitable to time-lapse imaging.

Instrumentation. Multiphoton microscopy of Laurdan requires a laser scanning microscope capable of at least two-channel imaging. To achieve high excitation efficiency, an objective with a high numerical aperture is required. This can be oil immersion (when imaging fixed, mounted cells), glycerol immersion (when imaging organisms, such as zebrafish embryos, embedded in agarose) or water immersion (when imaging live cells). When imaging di-4-ANEPPDHQ with single-photon excitation, the same rules apply.

The microscope must also have the appropriate wavelength and type of laser. Multiphoton excitation is typically achieved using a femtosecond- or picosecond-pulsed mode-locked laser, of which the most popular are titanium-sapphire lasers. For Laurdan, the laser is tuned to emit at 800 nm and is coupled into the external input port of the microscope. It is important that the microscope has the appropriate dichroic mirrors and filters to excite the sample at this wavelength. Di-4-ANEPPDHQ can be conveniently excited in the blue region of the spectrum from around 440–500 nm. This means that it can be used with the 488-nm line of common Argon ion lasers.

Similarly, for detection, the microscope has to have appropriate filters or spectral selection to detect Laurdan or di-4-ANEPPDHQ fluorescence in the recommended wavelength windows. Detection is most conveniently achieved via internal photomultiplier tubes (PMTs) in both cases. Note that as Laurdan is used with a multiphoton microscope, it is also possible to use PMTs via nondescanned detection if available, which will result in an increase in fluorescence detection efficiency.

Sample and biological considerations. Laurdan is applicable to fixed- and live-cell imaging and shows little to no toxicity at the concentrations recommended here. Di-4-ANEPPDHQ is generally not applicable to fixed-cell imaging as it is readily lost from the plasma membrane during the washing steps. Whether performing live- or fixed-cell imaging, the microscope should be fitted with an appropriate heated chamber, as it is known that temperature fluctuations can affect not only cell viability but also bilayer order, phase behavior and the solvent relaxation process directly. If the cells are sufficiently robust, the medium should be exchanged for phenol red-free medium or PBS to reduce background.

Data acquisition and calibration. Laurdan and di-4-ANEPPDHQ data are typically displayed as pseudocolored GP images. These are calculated according to the following equations:

$$GP = \frac{I_{400-460} - GI_{470-530}}{I_{400-460} + GI_{470-530}} \text{ and } GP = \frac{I_{500-580} - GI_{620-750}}{I_{500-580} + GI_{620-750}} \qquad (1)$$

for Laurdan and di-4-ANEPPDHQ, respectively. Here I represents the intensity in each pixel in the image acquired in the indicated spectral channel (numbers are in nm) and G is the calibration factor.

An important part of the data acquisition is the recording of calibration images to calculate the G factor. The G factor is used in the GP value calculation to compensate for differences in the efficiency of collection in the two channels caused by, among other things, the use of different PMT gains between experiments. The calibration is performed by imaging the dyes in a standard solution under standard conditions such that the ratio of fluorescence in each channel should always be constant. The G factor is calculated according to the following equation:

$$G = \frac{GP_{ref} + GP_{ref}GP_{mes} - GP_{mes} - 1}{GP_{mes} + GP_{ref}GP_{mes} - GP_{ref} - 1} \qquad (2)$$

Here GP_{mes} is the GP value of the respective dye in pure dimethyl sulfoxide (DMSO) measured with the same microscope setup and

© 2011 Nature America, Inc. All rights reserved.

settings as those used for the real sample. GP_{ref} is a reference value for the dye in DMSO, which can, in principle, be of any value but should always be held constant (otherwise, measurements from different experiments cannot be directly compared). A value for the GP of the dye in this standard sample is selected and is correct by definition. For Laurdan, the convention in the literature is that the reference GP value is $GP_{ref} = 0.207$ (ref. 45). This reference value was chosen so that the GP values for model membranes with liquid-ordered and -disordered phases are separated at around GP = 0. There is as yet no convention in the literature for the reference GP of di-4-ANEPPDHQ in DMSO, and the researcher is advised to choose a reference value that centers the GP histograms of cell data around GP = 0. For the imaging of cell membranes, this is typically achieved with a GP_{ref} for di-4-ANEPPDHQ of −0.85. In general, when new dyes are developed, the GP_{ref} value should also be selected by the user to center the GP histograms of cell images on GP = 0.

Typically, the raw input images should be thresholded so as to exclude any pixels with low intensity in either channel; i.e., background fluorescence. This ensures that later quantification only includes dye-stained membranes with a good signal-to-noise ratio. A good way to display the image data is to pseudo-color the grayscale image, for example, using a rainbow RGB (blue-red) lookup table (LUT). Thresholded pixels can then be colored black. The color range can be set to any value (e.g., GP = 0.5 = red, GP = −0.1 = blue) to maximize contrast. Further, it is possible to merge the pseudocolored pixels with the mean intensity image using the hue-saturation-brightness (HSB) color space. Here the GP value is assigned to hue (color), the mean intensity is set to brightness and saturation is set to 1. This has the effect of de-emphasizing dim (low signal-to-noise) pixels and producing an image that has both order (GP) and structural (intensity) information.

Software. The calculation of the GP images can be performed in a number of software packages such as MATLAB (MathWorks), LabVIEW (National Instruments) or ImageJ (National Institutes of Health) among others. Here we provide a custom-written macro for ImageJ (**Supplementary Data 1**).

MATERIALS

REAGENTS

- Laurdan or di-4-ANEPPDHQ (Molecular Probes, cat. no. D250; cat. no. D36802)
- DMSO (Sigma-Aldrich, cat. no. 472301-100ML)
- OPTIONAL: Paraformaldehyde 16% (wt/vol) solution (EM grade; Electron Microscopy Sciences, cat. no. 15710)
- Double-distilled water (ddH_2O)

For cell imaging

- Cultured HeLa cells (ATCC, cat. no. CCL-2)
- Dulbecco's modified Eagle's medium (DMEM low glucose, 1×; Gibco, Invitrogen, cat. no. 10567-014)
- PBS (Gibco, Invitrogen, cat. no. 14190-144)

For zebrafish imaging

- Zebrafish (*Danio rerio*) embryos **! CAUTION** Appropriate institutional ethics approval must be obtained and guidelines followed for the use of animals for research purposes
- Ethyl-3-aminobenzoate methanesulfonate (tricaine; Sigma-Aldrich, cat. no. E10521)
- Tris buffer
- PTU (1-phenyl-2-thiourea; Sigma-Aldrich, cat. no. 222909)
- Low melting agarose (1%, Sigma-Aldrich, cat. no. A9414)
- Sodium chloride (NaCl; Sigma-Aldrich, cat. no. S7653)
- Potassium chloride (KCl; Sigma-Aldrich, cat. no. P9333)
- Calcium chloride ($CaCl_2$; Sigma-Aldrich, cat. no. C1016)
- Magnesium sulfate ($MgSO_4$; Sigma-Aldrich, cat. no. M2643)
- Methylene blue (Sigma-Aldrich, cat. no. 28514)

EQUIPMENT

- Pasteur pipettes
- Microcentrifuge tubes (Eppendorf)
- TCS SP5 laser scanning inverted confocal microscope with Leica LAS software (Leica) **▲ CRITICAL** Other confocal microscopes, as well as multichannel widefield and TIRF microscopes can be used, as described in the Experimental design section.
- Microscope objectives: ×63 water objective (for live cells in medium), ×63 oil-immersion objective (for fixed, mounted cells) or a ×63 glycerol objective (for zebrafish in agarose)
- Mai-Tai mode-locked titanium-sapphire (Ti:Sapphire) laser (Spectra-Physics) **▲ CRITICAL** A mode-locked infrared laser is only required for Laurdan, and not for di-4-ANEPPDHQ. Laurdan can also be excited at 405 nm. Other mode-locked infrared lasers can also be used.
- Argon ion laser at 488 nm **▲ CRITICAL** Other lasers in the range of 440–500 nm can be used to excite di-4-ANEPPDHQ such as common 473 nm DPSSLs.
- Helium-neon (HeNe) laser (633 nm)
- Glass-bottomed microscope dishes with no. 1.5 thickness glass (Fluorodish, Coherent Scientific, cat. no. FD35-100)
- ImageJ (http://rsbweb.nih.gov/ij/) with Laurdan/di-4-ANEPPDHQ analysis macro installed (**Supplementary Data 1**) **▲ CRITICAL** Analysis software can also be written in other programming languages.

REAGENT SETUP

Dye stock solution Prepare a 5 mM stock solution of Laurdan or di-4-ANEPPDHQ in DMSO. This can be stored sealed in an airtight, lightproof glass vial at room temperature (21 °C) for up to 6 months.

Cells HeLa cells should be cultured in a glass-bottomed microscope dish at 37 °C in a humidified 5% CO_2 atmosphere such that they are approximately 40–70% confluent on the day of imaging. At this point, serum-free DMEM should be prewarmed to 37 °C.

Tricaine stock, 0.04% (wt/vol) Mix 400 mg of tricaine powder, 97.9 ml of double-distilled water and 2.1 ml of 1 M Tris buffer. Adjust the pH to 7. The solution can be stored in the freezer at −20 °C for several weeks. **! CAUTION** Tricaine is irritating to the eyes, respiratory system and skin, and thus contact should be avoided.

Stock solution of PTU Mix 150 mg of PTU in 100 ml of double-distilled water. The stock can be stored in a cool, dry area for several weeks. **! CAUTION** PTU is highly toxic, and thus contact should be avoided; use appropriate personal protective equipment.

E3 medium Mix 5 mM NaCl, 0.17 mM KCl, 0.33 mM $CaCl_2$, 0.33 mM $MgSO_4$ and 0.00001% (wt/vol) methylene blue in water. E3 medium can be stored at room temperature for several weeks.

Mate and raise zebrafish embryos according to a standard protocol[46]. At 24 h.p.f. (hours post fertilization), place the fertilized eggs in E3 medium containing PTU at a final concentration of 0.2 mM and incubate them at 28 °C. This step prevents pigment development (melanization inhibition) in zebrafish embryos and facilitates imaging. Keep zebrafish embryos that are 2–4 d.p.f. (days post fertilization) at 28 °C before conducting the experiment.

EQUIPMENT SETUP

The microscope and lasers should be set up to perform two-channel multi-photon microscopy (if using Laurdan) or two-channel confocal microscopy (if using di-4-ANEPPDHQ), as described in Step 2 of the PROCEDURE.

© 2011 Nature America, Inc. All rights reserved. npg

PROCEDURE

Sample staining

1| Use option A for staining live cells with Laurdan or di-4-ANEPPDHQ, or option B for staining live zebrafish embryos with Laurdan.

(A) Laurdan or di-4-ANEPPDHQ staining of live HeLa cells ● TIMING 40 min

(i) Remove medium from the cell culture dish and replace with 2 ml of fresh, serum-free DMEM.

▲ CRITICAL STEP Di-4-ANEPPDHQ shows a marked increase in background fluorescence in the presence of serum.

(ii) Add 2 μl of Laurdan or di-4-ANEPPDHQ stock (1:1,000 dilution, 5 μM final concentration).

▲ CRITICAL STEP The exact concentration required will vary depending on the cell density, the presence of lipid droplets in cells and potentially the presence of serum in the medium, which will sequester the dye. Laurdan concentrations of up to 50 μM can be used to efficiently stain cells without affecting cell viability. If the cell images appear dim with the described microscope settings (less than values of ~150 of an 8-bit (0–255) image), the concentration should be increased.

(iii) Shake the dish gently to ensure good mixing.

(iv) Incubate at 37 °C in a humidified 5% CO_2 atmosphere for 30 min.

▲ CRITICAL STEP Thirty minutes is the minimum time required for the dye to equilibrate between the different cellular membranes. Cells can be incubated with dye for several hours or overnight if required without substantial toxic effect.

(v) (Optional) At this point, the cells can be fixed if required. To fix, remove medium, wash with PBS and incubate in 4% (wt/vol) paraformaldehyde solution for 15 min at 37 °C in a humidified 5% CO_2 atmosphere.

▲ CRITICAL STEP Note that cold methanol fixation and glutaraldehyde fixation are unsuitable. Methanol extracts lipids and therefore influences membrane order; glutaraldehyde induces high background fluorescence.

▲ CRITICAL STEP Do not fix cells when using di-4-ANEPPDHQ, as the washing stages markedly decrease the di-4-ANEPPDHQ concentration in the plasma membrane.

(B) Laurdan staining in living zebrafish embryos ● TIMING 70 min

(i) Transfer 1 ml of E3 medium into a small Petri dish.

(ii) Add 5 or 10 μl of 5 mM Laurdan to obtain a 25 or 50 μM Laurdan concentration, respectively (the optimal concentration will depend on the age and number of embryos being stained). If the cell images appear dim with the described microscope settings (less than values of ~150 of an 8-bit (0–255) image), the concentration should be increased.

(iii) Transfer the embryos using a plastic Pasteur pipette to the Laurdan-containing E3 solution.

(iv) Incubate for 30 min at 28 °C (incubate 5 d.p.f. embryos for 1 h; note that 6 d.p.f. embryos are generally too large for suitable staining).

(v) Wash the embryos with PBS three times and allow them to recover for 30 min.

(vi) Meanwhile, prepare a 1% (wt/vol) low-melting agarose gel with E3 medium by heating in a boiling water bath and maintaining at 37–42 °C in a water bath to prevent solidification.

(vii) Transfer the embryos to a microcentrifuge tube and gently remove the E3 medium.

(viii) Add 50 μl of 0.04% (wt/vol) tricaine in water (which functions as an anesthetic) to the microcentrifuge tube.

(ix) Add 1 ml of agarose to the microcentrifuge tube and mix gently with the tricaine–E3 medium.

(x) Without delay, gently transfer the whole mixture into a microscope dish.

(xi) Position the embryos at the appropriate angle in the agarose to view the tissues of interest using a fine pin (0.10 mm diameter) mounted on a needle holder or on an applicator stick.

(xii) Allow the agarose to solidify over several minutes.

Imaging equipment setup ● TIMING 10 min

2| Use option A when imaging Laurdan in cells or zebrafish, or use option B for di-4-ANEPPDHQ imaging.

(A) Equipment setup for Laurdan imaging of cells or zebrafish embryos

(i) Turn on the microscope, scanner, Ti:Sapphire laser, 633-nm Helium-Neon (HeNe) laser, stage heater and the microscope computer and software (**Fig. 2a**, circle 1 shows correct laser activation in software). Note that other laser lines are acceptable to acquire a transmitted light image; e.g., a 561 nm DPSSL.

(ii) Tune the Ti:Sapphire laser to 800 nm (**Fig. 2b**, circle 2), ensure that it is mode locked (pulsing; **Fig. 2b**, circle 3) and open the laser shutter (**Fig. 2b**, circle 4).

(iii) Set the laser power to a low to moderate level (as shown in **Fig. 3**, circle 1).

▲ CRITICAL STEP Lower laser powers are advisable to avoid substantial photodamage of cells or tissues (**Fig. 3**, circle 1).

▲ CRITICAL STEP Note that if a transmitted light image is required, a visible laser (such as 561 or 633 nm) can be used to provide a signal detectable by the transmitted light detectors (**Fig. 3**, circle 2).

© 2011 Nature America, Inc. All rights reserved.
npg

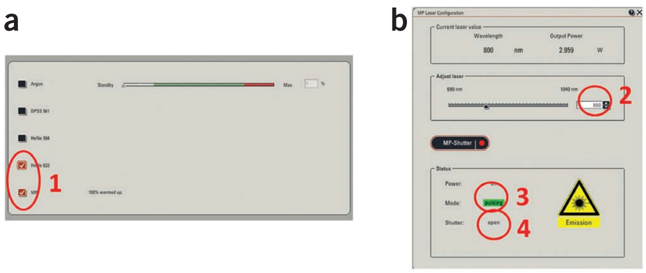

Figure 2 | Initialization of lasers for Laurdan microscopy in the Leica LAS software. (**a**) The multiphoton laser (MP) for Laurdan excitation and the 633-nm HeNe laser (to acquire a transmitted light image) are selected (circle 1). (**b**) The wavelength of the MP laser is set to 800 nm (circle 2). The laser is mode locked (pulsing; circle 3) and the MP laser shutter is open (circle 4).

(iv) Select a ×63 water objective (for live cells in medium), ×63 oil-immersion objective (for fixed, mounted cells) or a ×63 glycerol objective (for embryos in agarose; **Fig. 4**, circle 1).

▲ CRITICAL STEP Lower-magnification objectives can be used when imaging zebrafish to increase the field of view.

(v) Place a drop of ultrapure water, oil or glycerol on the lens.

(vi) Place the glass-bottomed dish containing the stained sample from Step 1 on the microscope stage.

(vii) Activate two PMTs and set the gains to maximum (**Fig. 4**, circle 2).

(viii) Set the detection ranges of the PMTs to 400–460 nm and 470–530 nm (**Fig. 4**, circle 3).

(ix) Turn on the transmitted light detector and set the gain to a moderate level (~200 V; **Fig. 4**, circle 4).

(x) Set the image size to 512 × 512 pixels (**Fig. 5**, circle 1), the scan speed to 400 Hz (**Fig. 5**, circle 2; higher or lower scan frequencies can be used if necessary) and the zoom to 1 (**Fig. 5**, circle 3).

(xi) Set the line average to at least 4 (**Fig. 5**, circle 4).

(xii) Fully open the confocal pinhole (**Fig. 5**, circle 5).

▲ CRITICAL STEP Set the pinhole to size 1 Airy unit if acquiring immunofluorescence or fluorescent protein images. Because of photobleaching, the immunofluorescence/protein images should generally be acquired first.

(B) Equipment setup for di-4-ANEPPDHQ imaging in live cells

(i) Turn on the microscope, scanner, Argon ion laser, stage heater and the microscope computer and software.

(ii) Activate the Argon ion laser line at 488 nm and set the laser power to 20%.

(iii) Repeat Step 2A(iv–vii).

(iv) Set the detection ranges of the PMTs to 500–580 nm and 620–750 nm.

(v) Repeat Step 2A(ix–xi).

(vi) Set the pinhole to size 1 Airy unit.

Sample image acquisition ● TIMING ~1 h

3| Select continuous acquisition and focus on the sample.

4| Select an underflow-overflow LUT.

5| Adjust the offset such that the minimum pixel value is around zero (underflow will be pseudocolored green).

6| Adjust the PMT gains such that neither channel contains saturated pixels (these appear blue in the underflow-overflow LUT) and such that each channel has approximately the same intensity.

▲ CRITICAL STEP Any saturated pixels will result in the GP calculation giving erroneous results. If the experiment involves a treatment that will increase/decrease membrane order, this will cause an increase in the intensity in one channel and should be anticipated in the choice of PMT gains. Having equal intensities in each channel maximizes the dynamic range of the measurement.

7| Select an appropriate level of zoom.

8| Ensure that the pixel values are still not saturated.

▲ CRITICAL STEP As the technique involves a ratiometric measurement between two channels, the PMT gains must be kept constant within an experiment. If subsequent samples vary in intensity, the laser power can be adjusted, as this affects both channels equally.

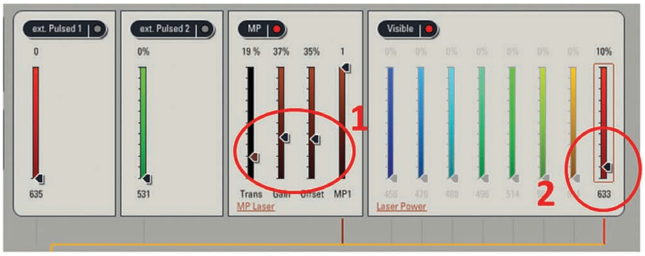

9| (Optional) Images can also be acquired in time-lapse or 3D imaging modes, or by using tiling, which is particularly useful when imaging embryos.

Figure 3 | Activation of lasers for Laurdan microscopy in the Leica LAS software. The MP laser is activated for Laurdan microscopy and the power level set to a moderate level (circle 1). The HeNe laser is activated to allow the capture of transmitted light images (circle 2).

© 2011 Nature America, Inc. All rights reserved.
npg

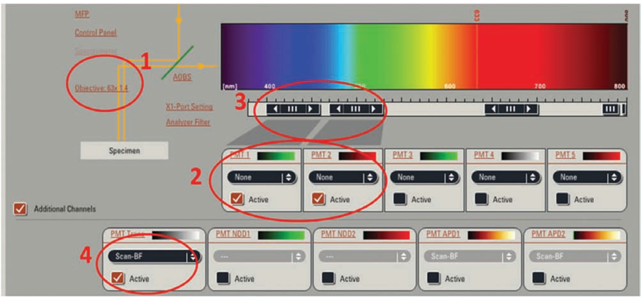

Figure 4 | Detection settings for Laurdan microscopy in the Leica LAS software. The microscope objective is set to a ×63 1.4 numerical aperture water-immersion lens (circle 1). Two PMTs are activated (circle 2). The detection wavelengths are set to 400–460 nm and 470–530 nm for PMT1 and PMT2, respectively (circle 3). Here PMT1 collects fluorescence from Laurdan primarily residing in the ordered phase. PMT2 collects fluorescence from Laurdan primarily residing in the disordered phase. The transmitted light detector (circle 4) is also activated.

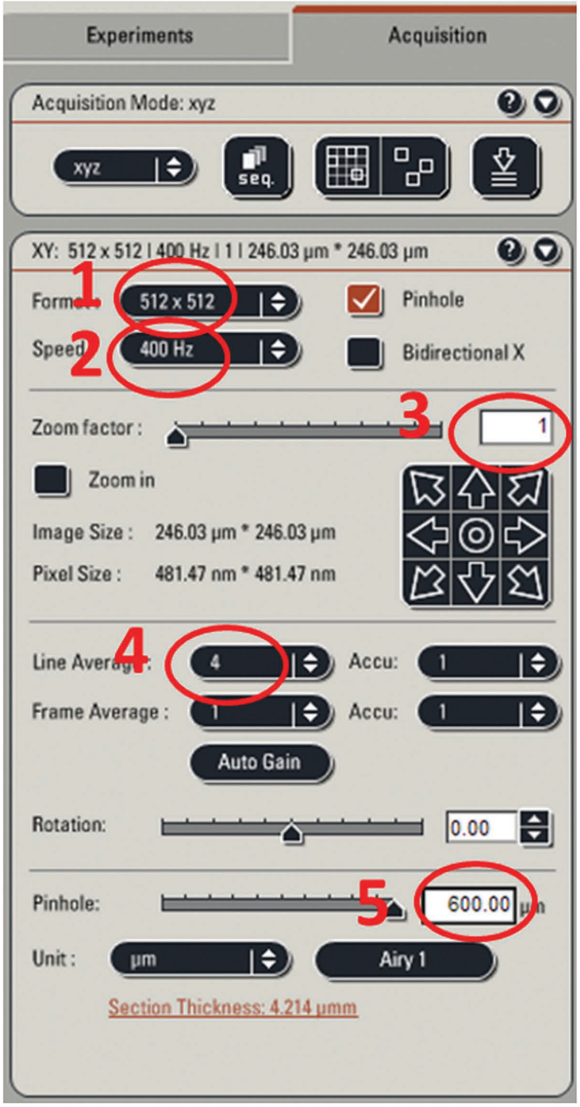

Figure 5 | Scan settings for Laurdan microscopy in the Leica LAS software. For Laurdan microscopy, images are typically acquired in 512 × 512 pixels (circle 1) with a scan speed of 400 Hz (circle 2). Initially, the zoom factor is set to 1 (circle 3). The line averaging should be set to at least 4 (circle 4) to improve the signal-to-noise ratio. As multiphoton microscopy is being used, the pinhole is fully opened to maximize light collection efficiency (circle 5).

10| Select 'Start' to acquire images.
? TROUBLESHOOTING

11| In the 'experiments' tab, right-click the data file and save the experiment as a .lif file.

12| Right-click and select 'Export to TIFF'.

13| Save the images as 8-bit grayscale .tiff format images (check the 'Save RAW data' box).
▲ **CRITICAL STEP** The channel file names will be saved as …ch00 (ordered phase) and …ch01 (disordered phase) such that they can be identified by the analysis program.

Calibration image acquisition ● TIMING ~15 min

14| Use instrument settings identical to those used for the experiment (i.e., objective, immersion media, PMT gains and offsets, zoom factor).
▲ **CRITICAL STEP** Do not change PMT settings as this will result in a false G factor and will therefore skew the GP values of cells/tissues toward higher/lower GP values.

15| Place an empty glass-bottomed dish on the microscope stage.

16| Add a ~1:100 dilution of the 5-mM Laurdan stock in DMSO, or add undiluted di-4-ANEPPDHQ stock to the dish (add around 5 µl total volume).

17| Focus on the dye solution.

18| Select continuous scan.

19| If the images are saturated (blue in the underflow-overflow LUT), dilute the dye solution further.

20| Record images with three different laser powers (the same power as that used for imaging the sample, as well as 50% higher power and 50% lower power).
▲ **CRITICAL STEP** Images must not be saturated. Therefore, the dye solution must be diluted further when using higher laser powers.

© 2011 Nature America, Inc. All rights reserved.
npg

21| Save images as .tiff files as described in Step 13.

Data analysis ● TIMING ~2 h

22| Open the ...ch00 (ordered) and ...ch01 (disordered) G-factor calibration images in ImageJ.
▲ **CRITICAL STEP** Other software can be used to determine the mean pixel intensities.

23| For each image, select 'Analyze' → 'Histogram' and record the mean value.

24| Calculate the G factor according to equation (2).

25| Repeat for G-factor images acquired at different laser powers and average the resulting G factors.

26| Close the calibration images.

27| Select 'Plugins' and select the provided macro (**Supplementary Data 1**) and a dialogue box will open. **Figure 6a** provides a flowchart of the macro's operation in generating the GP images, and **Figure 6b** shows the generation of pseudocolored GP-intensity merged images.
▲ **CRITICAL STEP** The analysis provided by the supplied macro can also be performed in other programming languages.

28| Navigate to the folder containing the saved images and click 'Open'.

29| Set the names of the ordered and disordered phase channels to ch00 and ch01, respectively (or the channel number corresponding to the ordered/disordered phase, respectively, if additionally acquiring differential interference contrast or immunofluorescence images; **Fig. 7a**, circle 1).

30| Set the threshold value for the analysis.
▲ **CRITICAL STEP** This operates on the mean of the two input images and should be set so as to not include dim pixels, as these will have low signal-to-noise ratios (default 15; **Fig. 7a**, circle 2).

31| Select the color scale for the output GP images.
▲ **CRITICAL STEP** This should be set to 'grays' if further quantification of the data is to be performed (**Fig. 7a**, circle 3).

32| Select 'no immunofluorescence mask' (**Fig. 7a**, circle 4).
▲ **CRITICAL STEP** Here it is possible to load a fluorescence mask image to define a region of interest (ROI; for example, the plasma membrane) for further analysis. If this is not used, ROIs can be selected manually later.

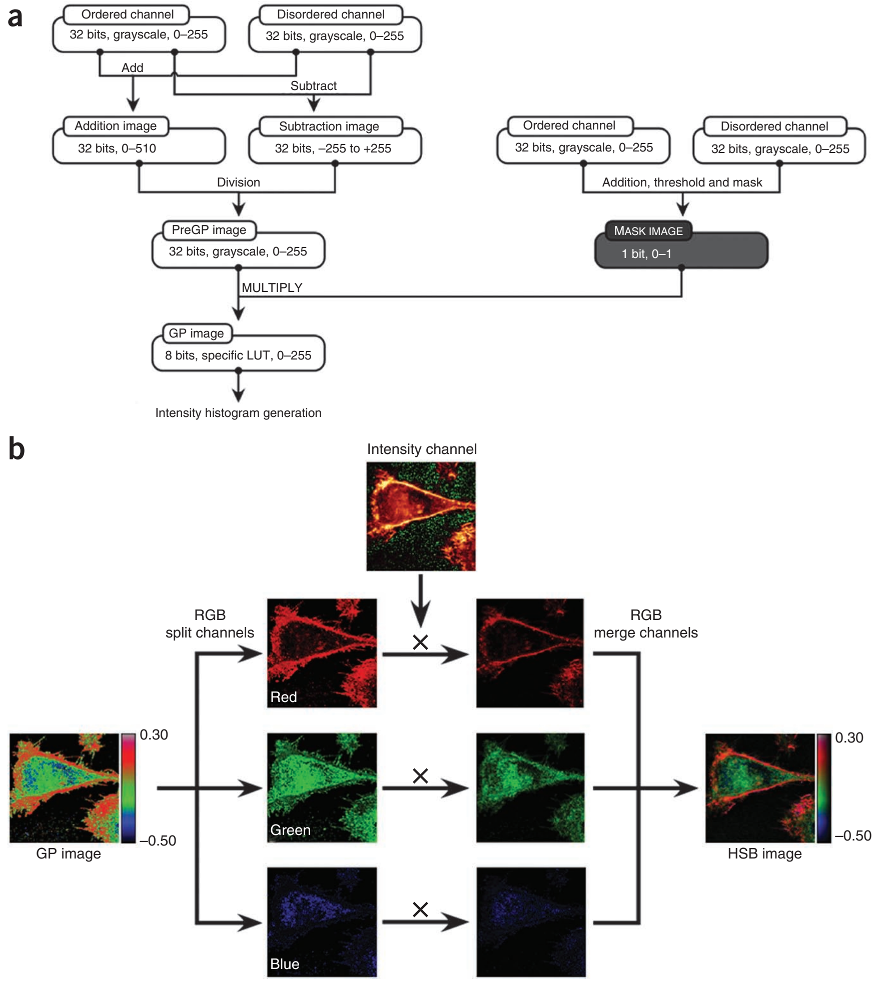

Figure 6 | Generating GP images and pseudocolored GP-intensity-merged images. (**a**) Flowchart of the operation of the GP analysis ImageJ macro for generating thresholded grayscale GP images from raw ordered and disordered channel images by following equation (1). (**b**) The principle of merging the pseudocolored GP image with the intensity image by multiplying each color component by the intensity value in that pixel.

© 2011 Nature America, Inc. All rights reserved.
npg

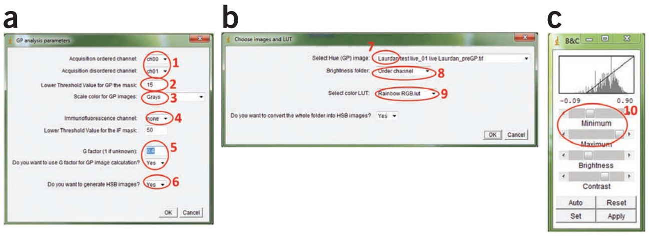

Figure 7 | Laurdan or di-4-ANEPPDHQ GP image processing. (**a**) The macro identifies the ordered and disordered phase channels by the file names, which should be set appropriately (circle 1). Typically, the images are thresholded before calculation of the GP values, as dim pixels have a poor signal-to-noise ratio. In this example, the threshold is set to 15 (circle 2). Two versions of the GP image are generated by the macro. The first is set to a grayscale image, which can be used for later quantification (circle 3). In this example, no immunofluorescence channel was acquired (circle 4). The G factor, calculated using equation (2) (Step 25), is set correctly (circle 5). The second type of image calculated by the macro is the pseudocolored, merged GP-intensity image (circle 6). (**b**,**c**) Generation of the merged image requires selecting a representative image (circle 7), the intensity image (circle 8), GP LUT (circle 9) (**b**) and the minimum and maximum GP values (circle 10) (**c**).

33| Set the value of the G factor to that calculated in equation 2 (Step 25). If the G factor was not calculated, set to 1 (default; **Fig. 7a**, circle 5).

34| Select whether or not to create merged GP-intensity images. These images are not used for quantification, and therefore the color scale can be selected to maximize contrast (rainbow RGB by default; **Fig. 7a**, circle 6).

35| Click 'OK', and if HSB images were selected, a new dialogue box will open.

36| Select a representative image from the drop-down list (**Fig. 7b**, circle 7). This will be used to set the GP color scale.

37| Select which intensity channel to merge with the GP image (**Fig. 7b**, circle 8).

38| Select a LUT for the GP values (**Fig. 7b**, circle 9).

39| Click 'OK'. The selected representative image will open with the GP values pseudocolored according to the selected LUT. The value of the minimum and maximum colors can now be set (**Fig. 7c**, circle 10). In this example, the minimum (blue) has been set to GP $= -0.09$. The maximum (red) has been set to GP $= 0.9$.

40| Click 'Apply' and 'OK'. The macro will process the images and create a new folder with the processed data. This contains the quantifiable grayscale GP images and extracted GP histograms from the whole images and the merged HSB GP-intensity images.
? TROUBLESHOOTING

41| Open a representative grayscale GP image in ImageJ.

42| Select an ROI using an appropriate drawing tool.

43| Select 'Analyze' → 'Histogram' and record the mean intensity value for that ROI. Copy and paste the histogram into a text file or Microsoft Excel and save.

44| Convert the 0:255 digital number (DN) values into $-1{:}+1$ GP values by using the formula GP $=$ DN / 127.5–1.

45| Normalize the histograms by dividing the number of pixels with each GP value by the total number of pixels in the ROI.

46| Plot the GP histograms. Disordered membranes will have histograms shifted to lower GP values. In cells, intracellular membranes are generally of lower order than the plasma membrane, for example.

? TROUBLESHOOTING
Troubleshooting advice can be found in **Table 1**.

© 2011 Nature America, Inc. All rights reserved.
npg

TABLE 1 | Troubleshooting table.

Step	Problem	Possible reason	Solution
10	Low plasma membrane staining	Cells were too dense or contain lipid droplets that have sequestered the dye	Decrease cell density (or zebrafish age) or increase the dye concentration and/or incubation time
	Low intracellular membrane staining	Cells were fixed or imaged too early after addition of the dyes	Increase the time between addition of the dye and imaging/fixation
	High background fluorescence	Cell media contains high levels of lipids or is relatively nonpolar	Swap full media for serum-free media or PBS. Alternatively, select a shorter maximum wavelength for the disordered channel (will also reduce GP image contrast)
40	No bright pixels, or background in the analyzed GP images	Threshold for GP calculation was set too high/low	Change the threshold used for GP calculation
	GP images have very high/low values or large areas of identical values	One or both of the channels was saturated during the acquisition or the channels were not set to similar intensities	Repeat the experiment with lower laser powers to prevent saturation<br>Adjust PMT settings to achieve similar intensities in both channels

© 2011 Nature America, Inc. All rights reserved.

● **TIMING**

Step 1, Staining: 30–40 min (cells), 60–80 min (zebrafish)
Step 2, Setup: 10 min
Steps 3–13, Acquisition of sample images: ~1 h
Steps 14–21, Acquisition of calibration images: ~15 min
Steps 22–46, Data analysis: ~2 h

ANTICIPATED RESULTS

Figure 8a shows a Laurdan GP image of muscle tissue acquired from the tail region of a 5-d-old zebrafish embryo together with the associated GP histogram (**Fig. 8b**). **Figure 9a** shows GP images of Laurdan-stained live HeLa cells, together with the associated GP histogram (**Fig. 9b**). **Figure 10a** shows GP images of di-4-ANEPPDHQ–stained live HeLa cells, together with the associated GP histogram (**Fig. 10b**). Note that in the cell examples (**Figs. 9** and **10**), the plasma membrane is higher ordered than the intracellular membranes. This is because the plasma membrane contains the majority of membrane cholesterol.

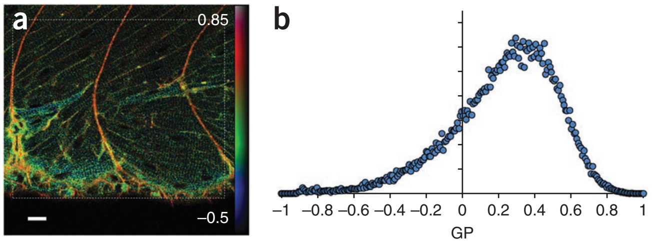

Figure 8 | Laurdan imaging of live zebrafish embryos. (**a**) Merged mean intensity image and rainbow RGB color GP image of muscle tissue in a live, 5-d-old zebrafish embryo tail. GP image is in false color and runs over the range indicated by the color bar. The image was acquired using Laurdan and multiphoton microscopy according to the PROCEDURE. Scale bar, 10 µm. (**b**) Histogram of the GP values from the region indicated by the white dashed box in **a**, normalized to the total number of pixels. The GP_{ref} value used was 0.207. Red colors in **a** indicate high membrane order, whereas blue colors indicate low order. The histogram (**b**) shows how many pixels have each GP value. Institutional regulatory board approval was obtained from the University of New South Wales Animal Care and Ethics Committee.

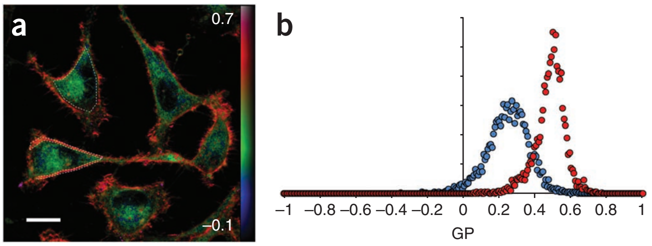

Figure 9 | Laurdan imaging of live HeLa cells. (**a**) Merged mean intensity image and rainbow RGB color GP image of live HeLa cells. GP image is in false color and runs over the range indicated by the color bar. The image was acquired using Laurdan and multiphoton microscopy according to the PROCEDURE. Scale bar, 10 µm. (**b**) Histogram of the GP values from the regions indicated by the white dashed boxes in **a** (blue, intracellular region; red, plasma membrane region), normalized to the total number of pixels. The GP_{ref} value used was 0.207. Red colors in **a** indicate high membrane order, whereas blue colors indicate low order. The histogram (**b**) shows how many pixels have each GP value. Note that the histogram for the plasma membrane is shifted to the right (higher GP), indicating higher membrane order than for intracellular membranes.

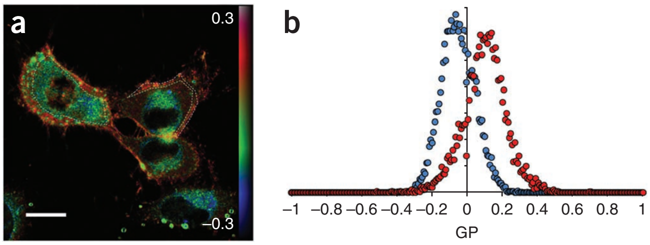

Figure 10 | Di-4-ANEPPDHQ imaging of live HeLa cells. (**a**) Merged mean intensity image and rainbow RGB color GP image of live HeLa cells. GP image is in false color and runs over the range indicated by the color bar. The image was acquired using di-4-ANEPPDHQ and confocal microscopy according to the PROCEDURE. Scale bar, 10 μm. (**b**) Histogram of the GP values from the regions indicated by the white dashed boxes in **a** (blue, intracellular region; red, plasma membrane region), normalized to the total number of pixels. The GP_{ref} value used was −0.85. Red colors in **a** indicate high membrane order, whereas blue colors indicate low order. The histogram (**b**) shows how many pixels have each GP value. Note that the histogram for the plasma membrane is shifted to the right (higher GP), indicating higher membrane order than for intracellular membranes.

Note: Supplementary information is available via the HTML version of this article.

ACKNOWLEDGMENTS D.M.O. and K.G. acknowledge funding from the Australian Research Council; K.G. also receives funding from the National Health and Medical Research Council of Australia and the Human Frontier Science Program. C.R. is grateful to the CONSOLIDER-INGENIO fellowship (CSD2009-00016, Ministerio de Innovación, Ciencia y Tecnología).

AUTHOR CONTRIBUTIONS D.M.O. developed the di-4-ANEPPDHQ protocol and wrote the manuscript. C.R. wrote the GP analysis macro. D.M.O. and A.M. conducted the experiments presented here. D.M.O., A.M. and A.A-S. developed the zebrafish protocol. K.G. developed the Laurdan protocol and wrote the manuscript.

COMPETING FINANCIAL INTERESTS The authors declare no competing financial interests.

Published online at http://www.natureprotocols.com/.
Reprints and permissions information is available online at http://www.nature.com/reprints/index.html.

© 2011 Nature America, Inc. All rights reserved.
npg

1. Simons, K. *et al.* Functional rafts in cell membranes. *Nature* **387**, 569–572 (1997).
2. Pike, L.J. Rafts defined: a report on the Keystone symposium on lipid rafts and cell function. *J. Lipid Res.* **47**, 1597–1598 (2006).
3. Tanimura, N. *et al.* Dynamic changes in the mobility of LAT in aggregated lipid rafts upon T cell activation. *J. Cell Biol.* **160**, 125–135 (2003).
4. Owen, D.M. *et al.* Quantitative microscopy: protein dynamics and membrane organisation. *Traffic* **10**, 962–971 (2009).
5. Gaus, K. *et al.* Condensation of the plasma membrane at the site of T lymphocyte activation. *J. Cell Biol.* **171**, 121–131 (2005).
6. Owen, D.M. *et al.* High plasma membrane lipid order imaged at the immunological synapse periphery in live T cells. *Mol. Membr. Biol.* **27**, 178–189 (2010).
7. Rentero, C. *et al.* Functional implications of plasma membrane condensation for T cell activation. *PLoS ONE* **3**, e2262 (2008).
8. Gupta, N. *et al.* Lipid rafts and B cell signaling. *Semin. Cell Dev. Biol.* **18**, 616–626 (2007).
9. Gaus, K. *et al.* Integrin-mediated adhesion regulates membrane order. *J. Cell Biol.* **174**, 725–734 (2006).
10. Barman, S. *et al.* Lipid raft disruption by cholesterol depletion enhances influenza A virus budding from MDCK cells. *J. Virol.* **81**, 12169–12178 (2007).
11. del Real, G. *et al.* Blocking of HIV-1 infection by targeting CD4 to nonraft membrane domains. *J. Exp. Med.* **196**, 293–301 (2002).
12. Scheiffele, P. *et al.* Influenza viruses select ordered lipid domains during budding from the plasma membrane. *J. Biol. Chem.* **274**, 2038–2044 (1999).
13. Ikonen, E. Roles of lipid rafts in membrane transport. *Curr. Opin. Cell Biol.* **13**, 470–477 (2001).
14. Hanzal-Bayer, M.F. *et al.* Lipid rafts and membrane traffic. *FEBS Lett.* **581**, 2098–2104 (2007).
15. Weber, G. & Farris, F.J. Synthesis and spectral properties of a hydrophobic fluorescent probe: 6-propionyl-2-(dimethylamino)naphthalene. *Biochemistry* **18**, 3075–3078 (1979).
16. Parasassi, T. *et al.* Laurdan and Prodan as polarity-sensitive fluorescent membrane probes. *J. Fluoresc.* **8**, 365–737 (1998).
17. Viard, M. *et al.* Laurdan solvatochromism: solvent dielectric relaxation and intramolecular excited-state reaction. *Biophys. J.* **73**, 2221–2234 (1997).
18. Vincent, M. *et al.* Nanosecond dynamics of a mimicked membrane-water interface observed by time-resolved stokes shift of LAURDAN. *Biophys. J.* **88**, 4337–4350 (2005).
19. Parasassi, T. *et al.* Phase fluctuation in phospholipid membranes revealed by Laurdan fluorescence. *Biophys. J.* **57**, 1179–1186 (1990).
20. Parasassi, T. *et al.* Two-photon fluorescence microscopy of laurdan generalized polarization domains in model and natural membranes. *Biophys. J.* **72**, 2413–2429 (1997).
21. Owen, D.M. *et al.* Imaging membrane lipid order in whole, living vertebrate organisms. *Biophys. J.* **99**, L7–L9 (2010).
22. Yu, W. *et al.* Fluorescence generalized polarization of cell membranes: a two-photon scanning microscopy approach. *Biophys. J.* **70**, 626–636 (1996).
23. Gaus, K. *et al.* Visualizing lipid structure and raft domains in living cells with two-photon microscopy. *Proc. Natl. Acad. Sci. USA* **100**, 15554–15559 (2003).
24. Gaus, K. *et al.* Condensation of the plasma membrane at the site of T lymphocyte activation. *J. Cell Biol.* **171**, 121–131 (2005).
25. Kaiser, H. *et al.* Order of lipid phases in model and plasma membranes. *Proc. Natl. Acad. Sci. USA* **106**, 16645–16650 (2009).
26. Römer, W. *et al.* Shiga toxin induces tubular membrane invaginations for its uptake into cells. *Nature* **450**, 670–675 (2007).
27. Römer, W. *et al.* Actin dynamics drive membrane reorganization and scission in clathrin-independent endocytosis. *Cell* **140**, 540–553 (2010).
28. Obaid, A.L. *et al.* Novel naphthylstyryl-pyridinium potentiometric dyes offer advantages for neural network analysis. *J. Neurosci. Meth.* **134**, 179–190 (2004).
29. Jin, L. *et al.* Cholesterol-enriched lipid domains can be visualized by di-4-ANEPPDHQ with linear and nonlinear optics. *Biophys. J.* **89**, L04–L06 (2005).
30. Jin, L. *et al.* Characterization and application of a new optical probe for membrane lipid domains. *Biophys. J.* **90**, 2563–2575 (2006).
31. Dinic, J. *et al.* Laurdan and di-4-ANEPPDHQ do not respond to membrane-inserted peptides and are good probes for lipid packing. *Biochim. Biophys. Acta* **1808**, 298–306 (2011).
32. Janes, P.W. *et al.* Aggregation of lipid rafts accompanies signaling via the T cell antigen receptor. *J. Cell Biol.* **147**, 447–461 (1999).
33. Zhang, R.-G. *et al.* The three-dimensional crystal structure of cholera toxin. *J. Mol. Biol.* **251**, 563–573 (1995).
34. Eggeling, C. *et al.* Direct observation of the nanoscale dynamics of membrane lipids in a living cell. *Nature* **457**, 1159–1163 (2009).
35. Wawrezinieck, L. *et al.* Fluorescence correlation spectroscopy diffusion laws to probe the submicron cell membrane organization. *Biophys. J.* **89**, 4029–4042 (2005).
36. Bacia, K. *et al.* Fluorescence correlation spectroscopy relates rafts in model and native membranes. *Biophys. J.* **87**, 1034–1043 (2004).
37. Kahya, N. *et al.* Probing lipid mobility of raft-exhibiting model membranes by fluorescence correlation spectroscopy. *J. Biol. Chem.* **278**, 28109–28115 (2003).
38. Lommerse, P.H.M. *et al.* Single-molecule diffusion reveals similar mobility for the Lck, H-Ras, and K-Ras membrane anchors. *Biophys. J.* **91**, 1090–1097 (2006).
39. Schneckenburger, H. *et al.* Time-gated total internal reflection fluorescence spectroscopy (TG-TIRFS): application to the membrane marker laurdan. *J. Microsc.* **211**, 30–36 (2003).

40. Grant, D.M. *et al.* High speed optically sectioned fluorescence lifetime imaging permits study of live cell signaling events. *Opt. Express* **15**, 15656–15673 (2007).
41. Miguel, L. *et al.* Primary human CD4+ T cells have diverse levels of membrane lipid order that correlate with their function. *J. Immunol.* **186**, 3505–3516 (2011).
42. Kim, H.M. *et al.* A two-photon fluorescent probe for lipid raft imaging: C-Laurdan. *Chembiochem* **8**, 553–559 (2007).
43. Kim, H.M. *et al.* Two-photon fluorescent turn-on probe for lipid rafts in live cell and tissue. *J. Am. Chem. Soc.* **130**, 4246–4247 (2008).
44. Veatch, S.L. *et al.* Fluorescent probes alter miscibility phase boundaries in ternary vesicles. *J. Phys. Chem.* **111**, 502–504 (2007).
45. Gaus, K. *et al.* Visualizing membrane microdomains by Laurdan 2-photon microscopy. *Mol. Membr. Biol.* **23**, 41–48 (2006).
46. Westerfield, M. *The Zebrafish Book: A Guide For the Laboratory Use of Zebrafish (Danio rerio)* 4th edn. (University of Oregon Press, 2000).

© 2011 Nature America, Inc. All rights reserved.

View publication stats
